# Identification of functional modules using network topology and high-throughput data

**DOI:** 10.1186/1752-0509-1-8

**Published:** 2007-01-26

**Authors:** Igor Ulitsky, Ron Shamir

**Affiliations:** 1 School of Computer Science, Tel Aviv University, Tel Aviv 69978, Israel

## Abstract

**Background:**

With the advent of systems biology, biological knowledge is often represented today by networks. These include regulatory and metabolic networks, protein-protein interaction networks, and many others. At the same time, high-throughput genomics and proteomics techniques generate very large data sets, which require sophisticated computational analysis. Usually, separate and different analysis methodologies are applied to each of the two data types. An integrated investigation of network and high-throughput information together can improve the quality of the analysis by accounting simultaneously for topological network properties alongside intrinsic features of the high-throughput data.

**Results:**

We describe a novel algorithmic framework for this challenge. We first transform the high-throughput data into similarity values, (e.g., by computing pairwise similarity of gene expression patterns from microarray data). Then, given a network of genes or proteins and similarity values between some of them, we seek connected sub-networks (or modules) that manifest high similarity. We develop algorithms for this problem and evaluate their performance on the osmotic shock response network in *S. cerevisiae *and on the human cell cycle network. We demonstrate that focused, biologically meaningful and relevant functional modules are obtained. In comparison with extant algorithms, our approach has higher sensitivity and higher specificity.

**Conclusion:**

We have demonstrated that our method can accurately identify functional modules. Hence, it carries the promise to be highly useful in analysis of high throughput data.

## Background

The accumulation of large-scale interaction data on multiple organisms, such as protein-protein and protein-DNA interactions, requires novel computational techniques that will be able to analyze these data together with information collected through other means. Such methods should enable thorough dissection of the data, whose dimensions have already extended far beyond the scope that is amenable to traditional analysis and manual interpretation. An important class of such biological information can be represented in the form of similarity relations. Quantitative molecular data, such as mRNA expression profiles, are often analyzed in this context through clustering algorithms. Similarity between genes can also be defined on other levels, such as function [1] or transcription factor binding patterns [2].

Although many fruitful algorithmic approaches have been developed for dissection of network and similarity data separately, methods analyzing together both information sources hold much promise. Several works have established the interconnection between expression profile similarity and protein interactions [3,4]. To exploit this interconnection, pairwise gene expression similarities have been used together with other data sources for predicting pairwise protein interactions (e.g., [5]). Topological properties of interaction networks induced by genes active in different conditions were studied [6-9]. Several software tools allow the visual inspection of the clustering results in a network context [10]. However, ignoring the network information in the clustering process and using the rich and constantly growing network information solely for cluster evaluation seems suboptimal, as the network information can improve the cluster identification process. The prevalence of modularity in molecular cell biology has been widely recognized in the last decade. By *functional module *one typically means a group of cellular components and their interactions that can be attributed a specific biological function [11]. Several approaches sought modules by jointly analyzing network information with gene expression data. Initial works [12,13] proposed measures for scoring expression activity in metabolic pathways (e.g. KEGG database [14]) and complexes [15]. Vert and Kanehisa [16] used kernel methods to identify expression patterns that characterize gene sets matching pathways in a given network.

The *Co-clustering *methodology [17] uses a distance function that combines similarity of gene expression profiles with network topology. The network distance between two nodes is an edge-weighted version of their topological distance in the network. The expression distance is one minus the Pearson correlation between the expression patterns. The two distances are combined into a similarity score, and standard hierarchical algorithms [18] are used for clustering. While generally successful, this approach sometimes produces clusters corresponding to highly disconnected subnetworks, since the network is only used as one of the sources of distance information, without requiring connectivity.

Ideker *et al*. [19] introduced a successful algorithm for identification of *active subnetworks*, i.e., connected regions of the network that show significant changes in expression over a particular subset of the conditions. Unfortunately, this method can be used only when one has an activity p-value for every measurement, a situation which is rather uncommon. In addition, the method cannot handle pairwise gene similarity input. The same methodology was recently used in [20], utilizing shortest-path algorithms for module finding. Segal *et al*. [21] provided another interesting formulation of the integration problem, in which a module is expected to contain a significant portion of the possible interactions. A probabilistic graphical model was used to extract a prespecified number of modules from gene expression measurements combined with a protein interaction dataset.

In this study we seek functional modules by identifying connected subnetworks in the interaction data that exhibit high average internal similarity. We call such a module a *Jointly Active Connected Subnetwork (JACS)*. By imposing network topology constraints on clusters of expression data, the biological interpretation of the clusters becomes easier, and, as we shall see, one can detect weaker signals that were indistinguishable by extant methods.

We develop a novel computational method for efficient detection and analysis of JACSs, implemented in a program called MATISSE (Module Analysis via Topology of Interactions and Similarity SEts). The proposed methodology has a statistical basis, which allows confidence estimation of the results. The algorithm assumes no prior knowledge on the number of JACSs, and allows imposing constraints on their size. We do not require precalculation of the statistical significance of expression values. The methodology is general enough to suit any type of network data overlaid with pairwise similarities.

Our algorithm detects JACSs by identifying heavy subgraphs in an edge-weighted similarity graph while maintaining connectivity in the interaction network. By transforming edge weights to attain probabilistic meaning, we are actually seeking subnetworks of maximum likelihood. We show that this problem is computationally hard, devise several heuristic methods and analyze their practical performance.

When using gene expression similarity, analysis of known pathways in yeast has shown that only a fraction of the genes in a pathway are usually coherently regulated at the transcription level (and thus highly similar) [22]. Our method allows assignment of different priors to different genes, reflecting their probability to be regulated at the transcription level. We believe this is the first study to allow such flexibility. In addition, the goal of our approach is to detect non-overlapping JACSs rather than to partition all the genes into clusters.

We first evaluate the performance of our algorithm on synthetic data with planted modules, and verify its ability to recover planted modules with high accuracy. Then, we analyze two real systems for which large datasets are available: the osmotic shock response of *S. cerevisiae*, and the cell cycle in human HeLa cells. For *S. cerevisiae*, we compiled and carefully annotated from diverse sources a protein-protein and protein-DNA interaction network consisting of 6,230 nodes and 89,327 interactions. The performance of MATISSE is shown to exceed that of extant analysis schemes in terms of the ability to retrieve biologically relevant groups, as analyzed by four different annotation datasets. We identify specific subnetworks relevant to different processes that are known to be activated and repressed by the MAPK cascades following osmotic shock, such as ergosterol biosynthesis and pheromone response. In addition, we identify novel pathways, such as pyridoxine metabolism, as differentially expressed during osmotic shock. Detailed analysis shows that some of the involved processes can not be detected based on the expression data alone. The human network contains 9,135 nodes and 25,086 protein-protein interactions collected from several sources, including recently published studies [23,24]. Our analysis identifies subnetworks active in specific phases of the human cell cycle. These results underly the ability of our approach to provide novel, previously undetected biological insights. The inspection of "hubs" in the subnetworks delineated by MATISSE reveals key regulators of the cell cycle.

## Results and discussion

### A framework for detection of jointly active subnetworks

Let us first state our problem abstractly. We are given an undirected *constraint graph G*^*C *^= (*V, E*), a subset *V*_*sim *_⊆ *V *and a symmetric matrix *S *where *S*_*ij *_is the *similarity *between *v*_*i*_, *v*_*j *_∈ *V*_*sim*_. The goal is to find disjoint subsets *U*_1_, *U*_2_,..., *U*_*m *_⊆ *V *called *JACSs*, so that each JACS induces a connected subgraph in *G*^*C *^and contains elements that share high similarity values. We call the nodes in *V*_*sim *_*front nodes *and nodes in *V*\*V*_*sim*_* back nodes*.

**Figure 1:**
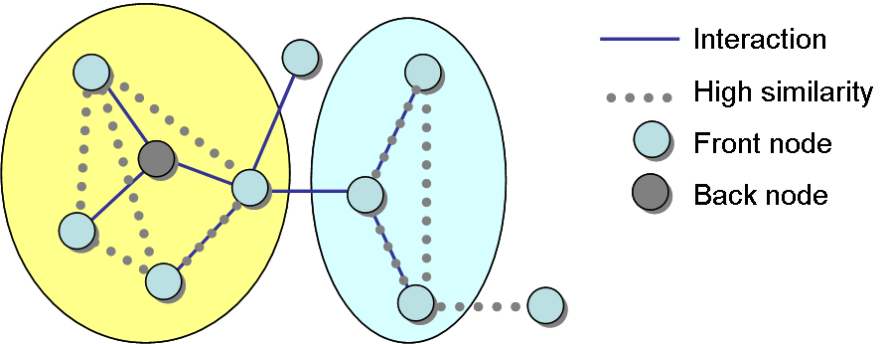
**Toy input example**. A toy example of an input problem with two distinct JACSs and with front and back nodes. Both JACSs (circled) are connected in the interaction network and heavy in the similarity graph. Note that the four front nodes in the left JACS form a connected subgraph only after the addition of the back node.

In the biological context, *V *represents genes or gene products (we shall use the term 'gene' for brevity), and *E *represents interactions between them. These can be known protein-protein or protein-DNA interactions or alternatively can originate from a known regulatory network where arc orientations are ignored. *S*_*ij *_measures the similarity between genes *i *and *j*, e.g., the Pearson correlation between their gene expression patterns. The set *V*_*sim *_may be smaller than *V *as some of the genes may be absent from the array, and others may show insignificant expression patterns across the tested conditions and thus excluded. Hence, a JACS aims to capture a set of genes that have highly similar behavior, and are also topologically connected, and thus may share a common function, e.g., belong to a single complex or pathway. As elaborated in Methods, we formulate the problem of JACS identification as a hypothesis testing question. In this approach statistically significant JACSs correspond to heavy subnetworks in a similarity graph, with nodes inducing a connected subgraph in *G*^*C *^(Figure 1). The probabilistic model we propose also accommodates the use of gene-specific priors, reflecting our confidence that they are transcriptionally regulated in the studied conditions.

As exact optimization is intractable, we designed and tested several heuristics for solving the problem (see Methods). The version that performed best on real biological data had the following three phases: (1) detection of relatively small, high-scoring gene sets, or *seeds*; for each node, the set consisting of it along with the neighboring nodes that are connected to it via positive-weighted edges was a candidate seed; (2) seed improvement, and (3) significance-based filtering (see Methods for full details). This version, which we call MATISSE, was used in subsequent analysis.

### Analysis of performance using simulated similarity values

In order to evaluate the ability of our method to detect subnetworks of high pairwise similarity, we first tested its performance on simulated similarity data. The simulation used a connected subnetwork of 2,000 nodes from the *S. cerevisiae *interaction network (described below) as the constraint graph. The similarity data were generated by "planting" a collection of JACSs with several defining parameters in the network, using two similarity value distributions, where members of the same JACS tend to have higher similarity, as described in Methods.

In order to test the effect of each parameter on the performance of the different module finding algorithms, we carried out simulations in which one parameter was varied while keeping the rest at their default values. We also tested simple clustering of the similarity data with the *K*-means algorithm and with the Co-clustering approach of Hanisch *et al*. [17], which proposes a distance measure based on topology and expression. Since the latter method does not readily provide clusters, we used that measure with a *K*-means-like algorithm (with *K *= 15, and moving genes between clusters based on average distance). Other methods (e.g., [21]) were not readily available for comparison.

**Figure 2:**
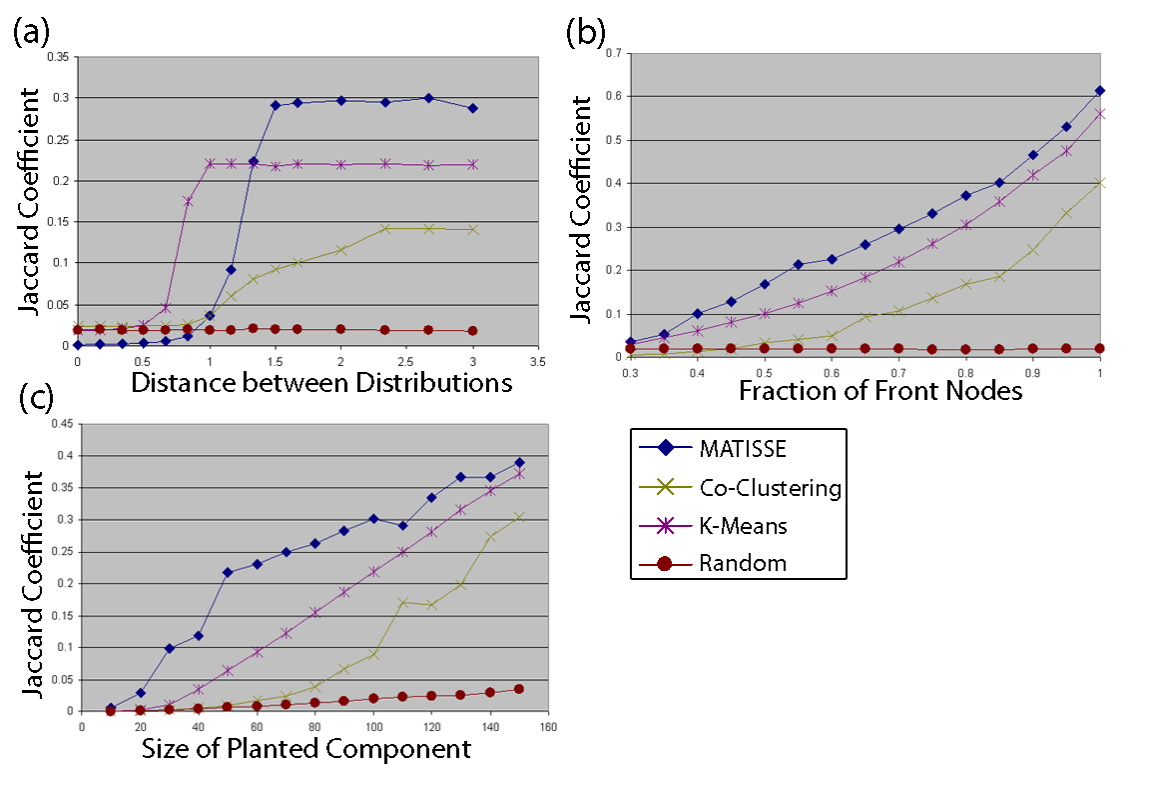
**Performance of different module finding procedures on simulated data**. Co-clustering: clustering based on the distance metric of [17]. K-Means: clustering of the similarity data. Random: random sampling of connected subnetworks matched in size and number to the planted components. The quality of solutions produced by the different procedures is evaluated by the Jaccard coefficient, (a) Performance as a function of the distance between the means of the mates and the non-mates distributions (*μ*_*m*_). (b) Performance as a function of the fraction of front nodes (*p*_*f*_). (c) Performance as a function of planted component size (*k*).

We evaluated the ability of the methods to recover the planted components using Jaccard coefficient. The coefficient ranges between 0 and 1 with 1 indicating perfect recovery (see Methods). The results are presented in Figure 2. MATISSE is able to retrieve the planted components with good precision when there is a plausible separation between the two similarity value distributions (above 1.3 standard deviations) and the fraction of the front nodes exceeds 0.8. The performance of MATISSE exceeds that of other methods for most of the parameter range.

### Response to osmotic stress in *S. cerevisiae*

We generated a comprehensive *S. cerevisiae *protein-protein and protein-DNA interaction network by combining information from the interaction databases SGD, BioGRID and BIND and recent high-throughput studies (e.g., [25], see our website for a complete list). This resulted in a network containing 6,230 nodes and 89,327 interactions. We also used 133 expression profiles of *S. cerevisiae *under different perturbations and different environmental conditions focused on the osmotic stress response [26]. The 2,000 genes whose patterns exhibit the highest variation in the data were designated as front nodes. We used Pearson correlation for scoring similarities between expression patterns. The parameters of the probabilistic model were assigned as described in Methods. Maps of the subnetworks produced by MATISSE are provided on our website and in the supplement [see Additional file 1].

#### Comparison of the modules produced by each method

We compared the performance of MATISSE to Co-clustering and to clustering based solely on the gene expression data. We used the CLICK algorithm [27] for clustering, as it was shown to outperform several extant gene expression clustering algorithms, and since it can determine the number of clusters and also leave some vertices unclustered. The Ideker *et al*. method [19] could not be tested in this setting, since measurement p-values could not be computed.

Table 1 compares the properties of the modules produced by every method. Expression homogeneity is calculated as the average Pearson correlation between genes within the same module. The *edge density *of a subgraph is the number of edges it contains as a fraction of all its node pairs. The *clustering coefficient *of a node is the fraction of its neighbor pairs that are connected in the network [28]. The clustering coefficient of a module is the average coefficient in the subgraph induced by the module. In the "Random connected" and "Random" solutions, modules were randomly sampled gene groups with and without the requirement for network connectivity, respectively. The sizes of the random groups were matched to the sizes obtained by MATISSE.

##### Expression homogeneity

As expected, the most homogeneous clusters in terms of expression similarity are obtained by CLICK, which optimized this type of similarity. The homogeneity of the MATISSE JACSs is higher than that of co-clusters. As previously reported [3], the expression homogeneity of a random connected set is higher than that of a random arbitrary set (average coherence of 0.063 for the random connected solution, vs. 0.033 for random arbitrary solution).

##### Topological descriptors

MATISSE is designed to produce connected subnetworks. The significance of this criterion is evident from the comparison to the other algorithms. In contrast to MATISSE, both CLICK and Co-clustering produce modules that are highly disconnected (averaging 80–90 components per module). Interestingly, the subnetworks produced by MATISSE are not denser than random connected components in the network. This observation can be explained by the fact that the network contains several dense complexes that do not participate in the solutions, as their components are not homogeneously expressed under the examined conditions.

#### Functional enrichment

In order to compare the functional relevance of the modules found by the different methods we used four annotation databases: (a) GO "biological process" ontology (level 7; 474 categories) [29]; (b) GO complexes annotation (subterms of "protein complex" term, 213 complexes); (c) MIPS deletion phenotype annotations [30] (181 phenotypes); (d) KEGG molecular pathways (310 pathways) [14]. A relatively wide selection of annotations was used to encompass diverse biological functions. Note that the GO "molecular function" categories are not relevant here, as the identified sets of genes are not expected to have similar molecular mechanisms.

**Figure 3:**
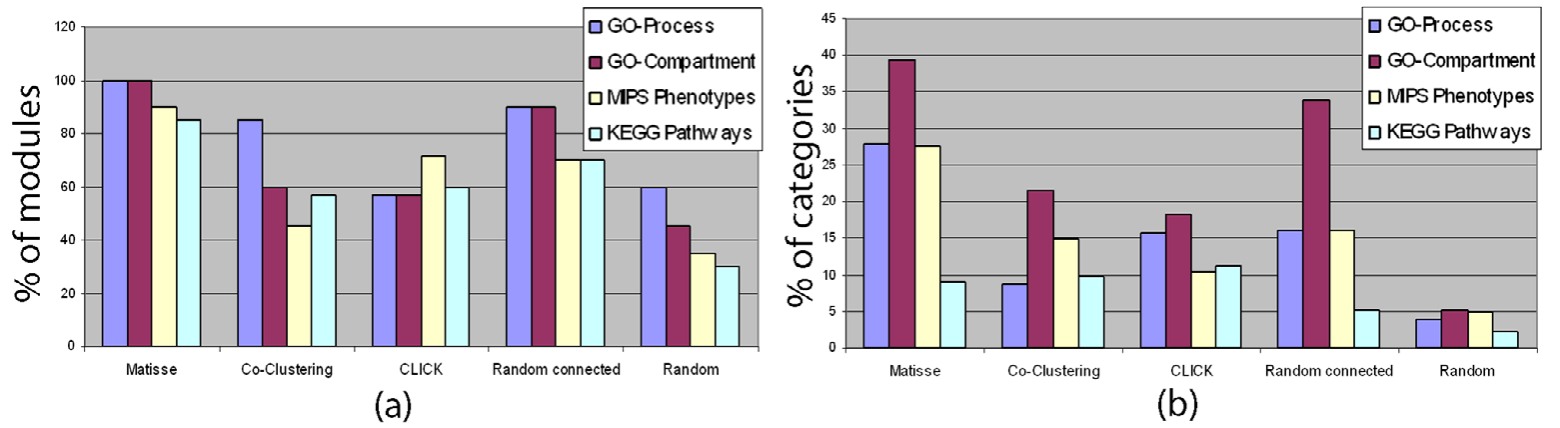
**Performance of different module finding algorithmson S. cerevisiae osmotic shock data**. (a) The fraction of the modules for which at least one category was enriched, (b) The fraction of the categories enriched in at least one module. Enrichment was defined as attaining hypergeometric p-value ≤ 10^-3^. Annotation sets: *GO-Process*: Level 7 of the GO "biological process" ontology; *GO-Complex*: subterms of "protein complex" term, GO:0043234; *MIPS Phenotypes*: MIPS deletion phenotype annotations; *KEGG Pathways*: KEGG molecular pathway participation.

For each annotation and for each group of genes produced by every method, the hypergeometric p-value was computed (without correcting for multiple testing, see below). We analyzed the percentage of the modules (Figure 3a) and of the categories (Figure 3b) enriched with p-value ≤ 10^-3 ^in each solution. MATISSE exhibits high performance in functional terms and in most cases the produced JACSs show higher enrichment than expression clusters and co-clusters. Co-clustering and CLICK perform slightly better than MATISSE in covering KEGG categories. This is probably due to the overrepresentation of metabolic pathways in KEGG. Metabolic pathways are generally poor in direct protein-protein and protein-DNA interactions, and thus less likely to be recognized by MATISSE, which relies also on direct interactions, than by a clustering algorithm based on expression alone.

As an additional comparison between MATISSE and Co-clustering, we compared the p-values obtained by each solution on each GO biological process (level 7) class attaining enrichment of *p *≤ 0.01 in at least one of the solutions. The MATISSE modules gave better significance to 238 functions, while only 116 functions had higher significance in the Co-clustering solution.

In order to check the added value of incorporating network constraints over using only expression profiles, we compared the results to clustering of the expression profiles with CLICK. In the same pairwise comparison, 223 MATISSE functions exhibited a higher enrichment, compared to 146 in CLICK. Several relevant functions, such as pyridoxine metabolism, cellular response to phosphate starvation, protein ubiquitination and post-Golgi transport, were enriched with *p *< 10^-5 ^in MATISSE, but were not significantly enriched in any CLICK cluster. When seeking functions enriched by the other clustering methods, the only function enriched was "NAD biosynthesis" (*p *< 10^-5^) discovered by CLICK. The six genes in our dataset that are annotated with this category do not contain any interactions between them and the average length of the shortest path between them is 7.

#### Functional subnetworks identified by MATISSE

In the previous analysis we did not correct for multiple testing since our goal was the comparison of the different methods. To address the multiple testing problem, we performed a GO functional enrichment analysis using the TANGO algorithm [31]. The algorithm considers all levels of the GO hierarchy and provides p-values corrected for multiple testing and for category dependency using resampling (see Methods).

21 distinct functional terms were found to be enriched (*p *< 0.05) in 14 distinct modules. The complete list of the enriched functions and their respective JACSs is shown in Table 2. Interactive maps of these JACSs can be found at our website along with the corresponding expression data. Note that JACSs were artificially limited to contain no more than 120 nodes in order to provide a better separation between pathways with slightly similar expression patterns. Nevertheless, it appears that this bound does not cause substantial fragmentation of the true clusters, as almost all the JACSs were enriched with distinct functions. Reassuringly, most of the enriched functions are highly relevant to the conditions and the perturbations in the data [32]. These include stress responses, such as repression of the translational machinery (JACSs 1–3) as well as general stress response genes (JACS 11 and 17). In addition, a specific subnetwork relevant to the activation of the pheromone response pathway following osmotic shock in *hog1 *strain [32] was identified (JACS 5). Indeed, since the HOG pathway shares protein kinases and phosphatases with other MAPK pathways, it was demonstrated that perturbations in Pbs2 or Hog1 lead to osmostress-induced stimulation of the pheromone response pathway [33].

JACS 7 contains seven genes from the yeast membrane ergosterol biosynthesis pathway which is strongly repressed following osmotic shock in the WT strain but not in *hog1 *strains. Lower levels of ergosterol make the membrane more compact and less flexible and hence lead to diminished transmembrane flux of glycerol, which is important for recovery from both hyper-osmotic and hypo-osmotic shock [32].

JACS 16 contains 19 genes members of the proteosome complex. 9 of these are back nodes, underlying the ability of MATISSE to use the network for linking co-activated genes with biologically relevant partners. Inspection of the expression data reveals a slight induction of the proteolysis genes following osmotic shock. This subtle response is missed when clustering solely the expression data, as no more than seven proteolysis genes are clustered together in the CLICK solution. Ubiquitin-dependent proteolytic mechanisms were linked to osmotic responses before [32], and our findings support this hypothesis.

**Figure 4:**
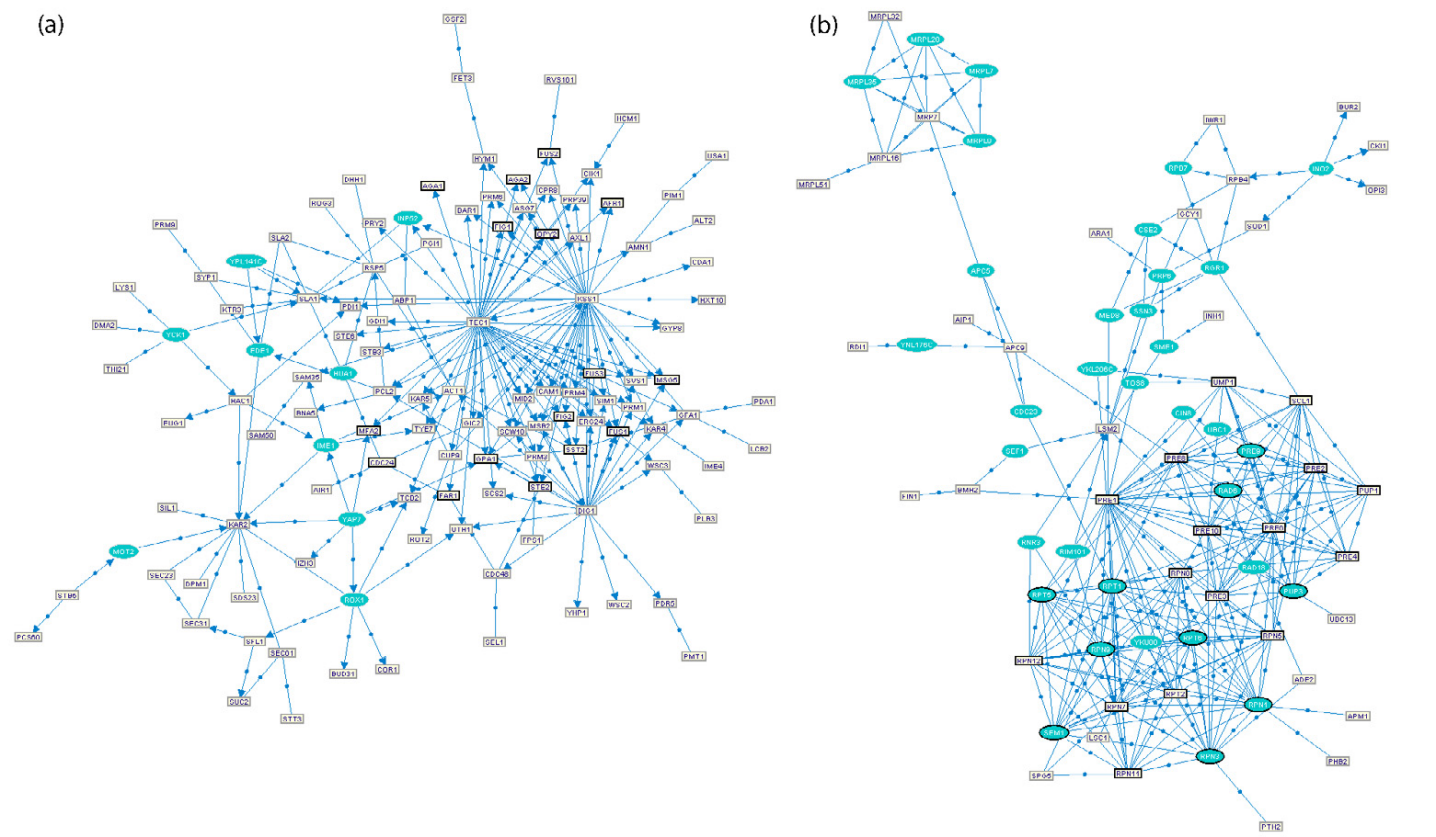
**Two of the JACSs identified in the S. cerevisiae analysis**. (a) The pheromone response subnetwork, (b) The proteolysis subnetwork. The front nodes are the yellow (light gray) rectangles and the back nodes and the blue (dark gray) ovals. The genes annotated with pheromone response (a) and proteolysis (b) are drawn with thicker border. Gene lists, expression matrices and interactive display of all the subnetworks are available at the supplementary website.

Figure 4 shows JACSs 5 and 16. These subnetworks demonstrate the use of different interaction types by MATISSE: JACS 5 is dominated by protein-DNA interactions, involving the transcription factors (TFs) Tec1, Kss1 and Dig1; JACS 16 is dominated by the protein interactions within the proteosome and the mitochondrial ribosome complexes. This subnetwork contains multiple back nodes linking front nodes. In fact, Table 2 shows that some JACSs make extensive use of nodes with no similarity data.

For several pathways, such as pyridoxine biosynthesis, intracellular transport and chromatin-related complexes (mainly SAGA, Cdc73, COMPASS and RSC) that were linked by MATISSE to osmotic shock in *S. cerevisiae*, this linking is novel. Pyridoxine was recently linked to osmotic shock response in *A. thaliana *[34]. These findings underlie the ability of MATISSE to produce testable hypotheses and novel insights.

#### Promoter analysis

Based on the assumption that genes that exhibit similar expression pattern over multiple conditions are likely to be co-regulated and to share common *cis*-regulatory elements in their promoters, we searched for over-representation of known transcription factor binding site motifs in the promoters of the genes in each JACS. When using the PRIMA motif finding tool [35], six subnetworks showed significant enrichment (*p *< 10^-5^) for at least one TF (Table 2). All the TFs corresponded to known regulators of the processes enriched in the subnetworks. For example, JACS 5, enriched for pheromone response pathway genes, was enriched with putative targets of Dig1 and Ste12, known regulators of these pathways [36]. Subnetwork 11, associated with general stress response, contained multiple targets of the Msn2 and Msn4 stress TFs [37]. We validated that these motif enrichments are not a byproduct of the functional enrichment in the JACSs (*p *< 10^-4^, by random sampling of gene groups with the same fraction of genes from the corresponding functional category as in the JACS). This analysis suggests that the JACS we obtained indeed correspond to gene modules with a common transcriptional regulation.

### Cell cycle in human

We constructed a human protein-protein interaction network by combining information from the BIND and HPRD databases and from two recent large-scale yeast two-hybrid studies on human cells [23,24]. The resulting network contains 9,135 nodes and 25,086 interactions. Expression profiles of the synchronized HeLa cell lines from [38] were used. Only the 19 point time series obtained for synchronization by thymidine-nocodazole block was selected for the analysis, as it contains the fewest missing values. Genes for which the maximal fold change across the conditions was below 2 were filtered, leaving 1,536 genes (front nodes).

**Figure 5:**
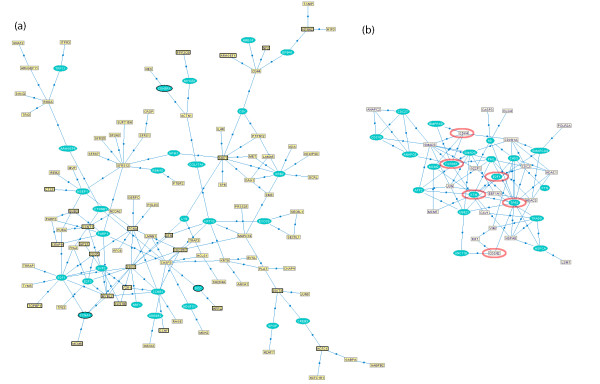
**Examples of the MATISSE analysis in the cell cycle data of human HeLa cells**. Front nodes and back nodes are as indicated in Figure 4. (a) The highest scoring cell-cycle related JACS identified. The genes annotated with "cell cycle" are drawn with thicker border. Gene lists, expression matrices and interactive display of all the subnetworks are available at the supplementary website, (b) Subnetwork hubs. The figure shows 36 nodes in the JACSs that were identified as subnetwork hubs and induced a connected component in the network. 16 additional hubs that had no interactions with other hubs are not shown. The known master regulators p53, ATM, E2F1, TGF*β*R, CDK4 and CDC42 are circled.

We performed MATISSE analysis using the All-Neighbors heuristic, and the same parameters as in the previous section, and obtained 14 significant JACSs. Maps of these subnetworks are provided on our website and in the supplement [see Additional file 1]. To check the ability to discover subnetworks active at different cell cycle phases, we analyzed the overlap between the JACSs and annotations of specific cell-cycle phases as provided in [38]. Indeed, seven modules were enriched for specific phases of the cell cycle with *p *< 0.05 after Bonferroni correction. The module with the highest cell cycle enrichment (JACS 5, *p *= 2.85·10^-17^) is shown in Figure 5a.

The advantage of MATISSE is evident when comparing the modules most enriched for the GO "cell cycle" category in the MATISSE and the Co-clustering solutions. While the MATISSE module is a single connected component of 120 genes, the corresponding co-cluster contains 110 connected components and 519 genes, and thus is much less amenable to interpretation in terms of the functional connections between its genes.

#### Subnetwork hub analysis

We hypothesized that the topology of the JACSs obtained by MATISSE can provide clues to the key players in the regulation of the cell cycle machinery. To test this, we looked for "subnetwork hubs" in the JACSs, i.e., genes whose degrees in a JACS were high both absolutely and relatively to their network degree (see Methods). This analysis on the 14 JACSs identified 52 hubs, 18 of them with "cell cycle" annotation (*p *= 5.13·10^-11^). This set contained many cell cycle master regulators such as p53, ATM, E2F1, TGF*β*R, CDK4 and CDC42. Remarkably, 36 out of 52 hubs form a single connected subnetwork, displayed in Figure 5b. This demonstrates that subnetwork hubs represent key regulators relevant to the experimental conditions tested. The interactions between the subnetwork hubs are putative regulatory interactions governing the progression of the cell cycle. As only 33 of the 52 hubs are front nodes in their respective JACS, this set could not be identified using expression data alone.

## Conclusion

We have developed a novel computational technique for the integrated analysis of network and similarity data. The method is aimed to dissect together topological properties of gene or protein networks and other high-throughput data. We used the method to analyze large-scale protein interaction networks and genome-wide transcription profiles in yeast and human. The method was shown to identify functionally sound modules, i.e., connected subnetworks with highly coherent expression showing significant functional enrichment. In comparison to the extant Co-clustering method, which aims to integrate similar data, our method demonstrated substantial improvement in solution quality. Comparison to solutions produced by clustering highlights the advantage of utilizing topological connectivity in the hunt for functionally sound modules. By construction, our method is specifically powerful in detection of regulatory modules, and less fit for detection of metabolic modules. Our technique, implemented in the program MATISSE, is efficient and can analyze genome-scale interaction and expression data within minutes.

The proposed algorithm is very flexible and – unlike Co-clustering – can handle situations where not all genes in the network have similarity information or expression patterns. In particular, MATISSE can determine the subset on which similarity is computed using various criteria, e.g., initial probe filtering, differential expression confidence values, etc. As we demonstrate, even when only a modest fraction of the overall network genes have expression/similarity information, the method finds meaningful modules successfully.

The requirement for network connectivity as proposed in our method can be viewed as problematic due to high rate of false negative interactions. A natural extension of MATISSE which we intend to pursue is to take into account the interaction confidence. As a first step towards this goal, we assessed the composition of the interactions in the reported subnetworks as follows: we compared the observed and expected number of interactions within the subnetworks, from each of the publications used as interaction sources in the *S. cerevisiae *interactions network. We found a clear enrichment for interactions from recent experiments, such as [39] and [40], opposed to an under-representation of interactions from older works, such as [41,42] and [43] (see supplementary table). As currently the coverage of the protein interaction network is limited, we suggest performing MATISSE analysis in addition to standard clustering analysis.

The framework described in this work is directly applicable to any kind of pairwise similarity data where the probabilistic assumptions hold. While this study focused on protein interaction networks and gene expression, the approach is general enough to treat many other data types. These include other types of interactions, such as genetic interactions, regulation and protein-DNA binding patterns, and other similarity measures, such as functional similarity or similarity in protein-DNA binding profiles [2]. We intend to extend MATISSE to these types of data as well.

While the rapidly expanding resource of microarray data is currently analyzed primarily using diverse clustering techniques, methods for the analysis of network-type data describing interrelations of genes and proteins are less mature, and methods for joint analysis of the two data types are in nascent stage. We expect the proposed method to become widely used for dissecting expression data in light of the interaction knowledge. Our initial results show that despite the high complexity and the relatively low coverage of the human interactome, biologically relevant modules can be found in the human protein interaction network through integrative analysis.

## Methods

### The probabilistic model

Recall that we formalize the problem as finding disjoint node sets that induce connected subgraphs in the constraint graph and manifest high internal similarity. We formulate this problem as a hypothesis testing question. For this, we define a probabilistic model for the similarity data, using ideas from [27] and [44]. Given a set *U *of *k *genes, we compare two hypotheses: the *null hypothesis H*_0_: *U *is a set of unrelated genes; and the *JACS hypothesis H*_1_: *U *is a JACS. We assume that the observed pairwise similarity values are a mixture of two Gaussian distributions: one for pairs of genes that are highly co-expressed (such pairs are called *mates*) and another for the rest. Let *M*_*ij *_denote the event that *i *and *j *are mates. The similarity values between mates (*P*(*S*_*ij*_|*M*_*ij*_)) are normally distributed with mean *μ*_*m *_and variance σm2
 MathType@MTEF@5@5@+=feaafiart1ev1aaatCvAUfKttLearuWrP9MDH5MBPbIqV92AaeXatLxBI9gBaebbnrfifHhDYfgasaacH8akY=wiFfYdH8Gipec8Eeeu0xXdbba9frFj0=OqFfea0dXdd9vqai=hGuQ8kuc9pgc9s8qqaq=dirpe0xb9q8qiLsFr0=vr0=vr0dc8meaabaqaciaacaGaaeqabaqabeGadaaakeaaiiGacqWFdpWCdaqhaaWcbaGaemyBa0gabaGaeGOmaidaaaaa@30F8@. The similarity levels of all non-mates are distributed normally with the parameters *μ*_*n *_and σn2
 MathType@MTEF@5@5@+=feaafiart1ev1aaatCvAUfKttLearuWrP9MDH5MBPbIqV92AaeXatLxBI9gBaebbnrfifHhDYfgasaacH8akY=wiFfYdH8Gipec8Eeeu0xXdbba9frFj0=OqFfea0dXdd9vqai=hGuQ8kuc9pgc9s8qqaq=dirpe0xb9q8qiLsFr0=vr0=vr0dc8meaabaqaciaacaGaaeqabaqabeGadaaakeaaiiGacqWFdpWCdaqhaaWcbaGaemOBa4gabaGaeGOmaidaaaaa@30FA@. These assumptions are theoretically justified in certain situations [27]. Empirically, analysis using normal quantile plots [45] indicates that they are valid for the biological data analyzed in this paper (results not shown). We also assume that the probability that a pair of genes are mates is high if they belong to the same JACS and low otherwise.

### Differential regulation

Not all genes within the interaction network are regulated on the expression level. Thus, when working with expression profiles, we would like the model to allow lower similarity levels between genes that are not necessarily regulated on the expression level, while penalizing heavily for low similarity between transcriptionally regulated genes. This allows flexibility on two levels in our setting. First, the genes can be filtered prior to computing similarities (e.g., only genes passing a threshold of observed fold change or variation level are included in *V*_*sim*_). Note that genes that fail to pass the filter remain in the interaction network and can be incorporated into a JACS, while not used for its scoring. Second, a prior can be assigned to the likelihood that a gene is regulated: we define *R*_*i *_as the event that gene *i *is regulated on the expression level under the conditions studied and let *P*(*R*_*i*_) designate the probability of that event.

### The likelihood score

We assume that JACSs contain a much higher proportion of mates than gene pairs that do not belong to the same JACS. Specifically, we assume that a large fraction *β*_*m *_(e.g. 0.9) of the pairs of transcriptionally regulated genes within the JACS are mates and thus their similarity levels are distributed *N*(*μ*_*m*_, *σ*_*m*_). Then *P*(*M*_*ij*_|*R*_*i *_∧ *R*_*j*_, *H*_1_) = *β*_*m*_. We make the simplifying approximation that the scores of different gene pairs are independent. Consequently, the likelihood of a JACS *U *is decomposable on every pair of genes in it:

P(SU×U|H1)=∏(i,j)∈U×UP(Sij|H1)
 MathType@MTEF@5@5@+=feaafiart1ev1aaatCvAUfKttLearuWrP9MDH5MBPbIqV92AaeXatLxBI9gBaebbnrfifHhDYfgasaacH8akY=wiFfYdH8Gipec8Eeeu0xXdbba9frFj0=OqFfea0dXdd9vqai=hGuQ8kuc9pgc9s8qqaq=dirpe0xb9q8qiLsFr0=vr0=vr0dc8meaabaqaciaacaGaaeqabaqabeGadaaakeaacqWGqbaucqGGOaakcqWGtbWudaWgaaWcbaGaemyvauLaey41aqRaemyvaufabeaakiabcYha8jabdIeainaaBaaaleaacqaIXaqmaeqaaOGaeiykaKIaeyypa0ZaaebuaeaacqWGqbaucqGGOaakcqWGtbWudaWgaaWcbaGaemyAaKMaemOAaOgabeaakiabcYha8jabdIeainaaBaaaleaacqaIXaqmaeqaaOGaeiykaKcaleaacqGGOaakcqWGPbqAcqGGSaalcqWGQbGAcqGGPaqkcqGHiiIZcqWGvbqvcqGHxdaTcqWGvbqvaeqaniabg+Givdaaaa@5242@

Let γijm
 MathType@MTEF@5@5@+=feaafiart1ev1aaatCvAUfKttLearuWrP9MDH5MBPbIqV92AaeXatLxBI9gBaebbnrfifHhDYfgasaacH8akY=wiFfYdH8Gipec8Eeeu0xXdbba9frFj0=OqFfea0dXdd9vqai=hGuQ8kuc9pgc9s8qqaq=dirpe0xb9q8qiLsFr0=vr0=vr0dc8meaabaqaciaacaGaaeqabaqabeGadaaakeaaiiGacqWFZoWzdaqhaaWcbaGaemyAaKMaemOAaOgabaGaemyBa0gaaaaa@32A2@ = *β*_*m*_*P*(*R*_*i*_)*P*(*R*_*j*_). Then:

*P*(*S*_*ij*_|*H*_1_) = γijm
 MathType@MTEF@5@5@+=feaafiart1ev1aaatCvAUfKttLearuWrP9MDH5MBPbIqV92AaeXatLxBI9gBaebbnrfifHhDYfgasaacH8akY=wiFfYdH8Gipec8Eeeu0xXdbba9frFj0=OqFfea0dXdd9vqai=hGuQ8kuc9pgc9s8qqaq=dirpe0xb9q8qiLsFr0=vr0=vr0dc8meaabaqaciaacaGaaeqabaqabeGadaaakeaaiiGacqWFZoWzdaqhaaWcbaGaemyAaKMaemOAaOgabaGaemyBa0gaaaaa@32A2@*P*(*S*_*ij*_|*M*_*ij*_) + (1 - γijm
 MathType@MTEF@5@5@+=feaafiart1ev1aaatCvAUfKttLearuWrP9MDH5MBPbIqV92AaeXatLxBI9gBaebbnrfifHhDYfgasaacH8akY=wiFfYdH8Gipec8Eeeu0xXdbba9frFj0=OqFfea0dXdd9vqai=hGuQ8kuc9pgc9s8qqaq=dirpe0xb9q8qiLsFr0=vr0=vr0dc8meaabaqaciaacaGaaeqabaqabeGadaaakeaaiiGacqWFZoWzdaqhaaWcbaGaemyAaKMaemOAaOgabaGaemyBa0gaaaaa@32A2@)*P*(*S*_*ij*_|Mij¯
 MathType@MTEF@5@5@+=feaafiart1ev1aaatCvAUfKttLearuWrP9MDH5MBPbIqV92AaeXatLxBI9gBaebbnrfifHhDYfgasaacH8akY=wiFfYdH8Gipec8Eeeu0xXdbba9frFj0=OqFfea0dXdd9vqai=hGuQ8kuc9pgc9s8qqaq=dirpe0xb9q8qiLsFr0=vr0=vr0dc8meaabaqaciaacaGaaeqabaqabeGadaaakeaadaqdaaqaaiabd2eannaaBaaaleaacqWGPbqAcqWGQbGAaeqaaaaaaaa@30C4@)

The null hypothesis (*H*_0_) is that the fraction of mates in *U *is not surprising: every two transcriptionally regulated genes are mates with the probability expected from the relative portion of mates among all the regulated genes, denoted *p*_*m*_. Let γijn
 MathType@MTEF@5@5@+=feaafiart1ev1aaatCvAUfKttLearuWrP9MDH5MBPbIqV92AaeXatLxBI9gBaebbnrfifHhDYfgasaacH8akY=wiFfYdH8Gipec8Eeeu0xXdbba9frFj0=OqFfea0dXdd9vqai=hGuQ8kuc9pgc9s8qqaq=dirpe0xb9q8qiLsFr0=vr0=vr0dc8meaabaqaciaacaGaaeqabaqabeGadaaakeaaiiGacqWFZoWzdaqhaaWcbaGaemyAaKMaemOAaOgabaGaemOBa4gaaaaa@32A4@ = *p*_*m*_*P*(*R*_*i*_)*P*(*R*_*j*_). The likelihood ratio between the two hypotheses (P(Data|H1)P(Data|H0))
 MathType@MTEF@5@5@+=feaafiart1ev1aaatCvAUfKttLearuWrP9MDH5MBPbIqV92AaeXatLxBI9gBaebbnrfifHhDYfgasaacH8akY=wiFfYdH8Gipec8Eeeu0xXdbba9frFj0=OqFfea0dXdd9vqai=hGuQ8kuc9pgc9s8qqaq=dirpe0xb9q8qiLsFr0=vr0=vr0dc8meaabaqaciaacaGaaeqabaqabeGadaaakeaacqGGOaakdaWcaaqaaiabdcfaqjabcIcaOiabdseaejabdggaHjabdsha0jabdggaHjabcYha8jabdIeainaaBaaaleaacqaIXaqmaeqaaOGaeiykaKcabaGaemiuaaLaeiikaGIaemiraqKaemyyaeMaemiDaqNaemyyaeMaeiiFaWNaemisaG0aaSbaaSqaaiabicdaWaqabaGccqGGPaqkaaGaeiykaKcaaa@45D0@ is:

∏(i,j)∈U×UγijmP(Sij|Mij)+(1−γijm)P(Sij|Mij¯)∏(i,j)∈U×UγijnP(Sij|Mij)+(1−γijn)P(Sij|Mij¯)=∏(i,j)∈U×UγijmP(Sij|Mij)+(1−γijm)P(Sij|Mij¯)γijnP(Sij|Mij)+(1−γijn)P(Sij|Mij¯)
MathType@MTEF@5@5@+=feaafiart1ev1aaatCvAUfKttLearuWrP9MDH5MBPbIqV92AaeXatLxBI9gBaebbnrfifHhDYfgasaacH8akY=wiFfYdH8Gipec8Eeeu0xXdbba9frFj0=OqFfea0dXdd9vqai=hGuQ8kuc9pgc9s8qqaq=dirpe0xb9q8qiLsFr0=vr0=vr0dc8meaabaqaciaacaGaaeqabaqabeGadaaakeaadaWcaaqaamaarababaacciGae83SdC2aa0baaSqaaiabdMgaPjabdQgaQbqaaiabd2gaTbaaaeaacqGGOaakcqWGPbqAcqGGSaalcqWGQbGAcqGGPaqkcqGHiiIZcqWGvbqvcqGHxdaTcqWGvbqvaeqaniabg+GivdGccqWGqbaucqGGOaakcqWGtbWudaWgaaWcbaGaemyAaKMaemOAaOgabeaakiabcYha8jabd2eannaaBaaaleaacqWGPbqAcqWGQbGAaeqaaOGaeiykaKIaey4kaSIaeiikaGIaeGymaeJaeyOeI0Iae83SdC2aa0baaSqaaiabdMgaPjabdQgaQbqaaiabd2gaTbaakiabcMcaPiabdcfaqjabcIcaOiabdofatnaaBaaaleaacqWGPbqAcqWGQbGAaeqaaOGaeiiFaW3aa0aaaeaacqWGnbqtdaWgaaWcbaGaemyAaKMaemOAaOgabeaaaaGccqGGPaqkaeaadaqeqaqaaiab=n7aNnaaDaaaleaacqWGPbqAcqWGQbGAaeaacqWGUbGBaaaabaGaeiikaGIaemyAaKMaeiilaWIaemOAaOMaeiykaKIaeyicI4SaemyvauLaey41aqRaemyvaufabeqdcqGHpis1aOGaemiuaaLaeiikaGIaem4uam1aaSbaaSqaaiabdMgaPjabdQgaQbqabaGccqGG8baFcqWGnbqtdaWgaaWcbaGaemyAaKMaemOAaOgabeaakiabcMcaPiabgUcaRiabcIcaOiabigdaXiabgkHiTiab=n7aNnaaDaaaleaacqWGPbqAcqWGQbGAaeaacqWGUbGBaaGccqGGPaqkcqWGqbaucqGGOaakcqWGtbWudaWgaaWcbaGaemyAaKMaemOAaOgabeaakiabcYha8naanaaabaGaemyta00aaSbaaSqaaiabdMgaPjabdQgaQbqabaaaaOGaeiykaKcaaiabg2da9maarafabaWaaSaaaeaacqWFZoWzdaqhaaWcbaGaemyAaKMaemOAaOgabaGaemyBa0gaaOGaemiuaaLaeiikaGIaem4uam1aaSbaaSqaaiabdMgaPjabdQgaQbqabaGccqGG8baFcqWGnbqtdaWgaaWcbaGaemyAaKMaemOAaOgabeaakiabcMcaPiabgUcaRiabcIcaOiabigdaXiabgkHiTiab=n7aNnaaDaaaleaacqWGPbqAcqWGQbGAaeaacqWGTbqBaaGccqGGPaqkcqWGqbaucqGGOaakcqWGtbWudaWgaaWcbaGaemyAaKMaemOAaOgabeaakiabcYha8naanaaabaGaemyta00aaSbaaSqaaiabdMgaPjabdQgaQbqabaaaaOGaeiykaKcabaGae83SdC2aa0baaSqaaiabdMgaPjabdQgaQbqaaiabd6gaUbaakiabdcfaqjabcIcaOiabdofatnaaBaaaleaacqWGPbqAcqWGQbGAaeqaaOGaeiiFaWNaemyta00aaSbaaSqaaiabdMgaPjabdQgaQbqabaGccqGGPaqkcqGHRaWkcqGGOaakcqaIXaqmcqGHsislcqWFZoWzdaqhaaWcbaGaemyAaKMaemOAaOgabaGaemOBa4gaaOGaeiykaKIaemiuaaLaeiikaGIaem4uam1aaSbaaSqaaiabdMgaPjabdQgaQbqabaGccqGG8baFdaqdaaqaaiabd2eannaaBaaaleaacqWGPbqAcqWGQbGAaeqaaaaakiabcMcaPaaaaSqaaiabcIcaOiabdMgaPjabcYcaSiabdQgaQjabcMcaPiabgIGiolabdwfavjabgEna0kabdwfavbqab0Gaey4dIunaaaa@FB57@

Define the *similarity graph*, *G*^*S *^= (*V*_*sim*_, *E*^*S*^), where *E*^*S *^= (*V*_*sim *_× *V*_*sim*_) and set wij=log⁡γijmP(Sij|Mij)+(1−γijm)P(Sij|Mij¯)γijnP(Sij|Mij)+(1−γijn)P(Sij|Mij¯)
 MathType@MTEF@5@5@+=feaafiart1ev1aaatCvAUfKttLearuWrP9MDH5MBPbIqV92AaeXatLxBI9gBaebbnrfifHhDYfgasaacH8akY=wiFfYdH8Gipec8Eeeu0xXdbba9frFj0=OqFfea0dXdd9vqai=hGuQ8kuc9pgc9s8qqaq=dirpe0xb9q8qiLsFr0=vr0=vr0dc8meaabaqaciaacaGaaeqabaqabeGadaaakeaacqWG3bWDdaWgaaWcbaGaemyAaKMaemOAaOgabeaakiabg2da9iGbcYgaSjabc+gaVjabcEgaNnaalaaabaacciGae83SdC2aa0baaSqaaiabdMgaPjabdQgaQbqaaiabd2gaTbaakiabdcfaqjabcIcaOiabdofatnaaBaaaleaacqWGPbqAcqWGQbGAaeqaaOGaeiiFaWNaemyta00aaSbaaSqaaiabdMgaPjabdQgaQbqabaGccqGGPaqkcqGHRaWkcqGGOaakcqaIXaqmcqGHsislcqWFZoWzdaqhaaWcbaGaemyAaKMaemOAaOgabaGaemyBa0gaaOGaeiykaKIaemiuaaLaeiikaGIaem4uam1aaSbaaSqaaiabdMgaPjabdQgaQbqabaGccqGG8baFdaqdaaqaaiabd2eannaaBaaaleaacqWGPbqAcqWGQbGAaeqaaaaakiabcMcaPaqaaiab=n7aNnaaDaaaleaacqWGPbqAcqWGQbGAaeaacqWGUbGBaaGccqWGqbaucqGGOaakcqWGtbWudaWgaaWcbaGaemyAaKMaemOAaOgabeaakiabcYha8jabd2eannaaBaaaleaacqWGPbqAcqWGQbGAaeqaaOGaeiykaKIaey4kaSIaeiikaGIaeGymaeJaeyOeI0Iae83SdC2aa0baaSqaaiabdMgaPjabdQgaQbqaaiabd6gaUbaakiabcMcaPiabdcfaqjabcIcaOiabdofatnaaBaaaleaacqWGPbqAcqWGQbGAaeqaaOGaeiiFaW3aa0aaaeaacqWGnbqtdaWgaaWcbaGaemyAaKMaemOAaOgabeaaaaGccqGGPaqkaaaaaa@894D@ as the weight of the edge (*v*_*i*_, *v*_*j*_). The log-likelihood score for a given *U *translates to the total edge weight of the subgraph induced by *U *in *G*^*S*^.

### JACS finding algorithm

Our goal is to find disjoint sets *U*_1_, *U*_2_,..., *U*_*m *_that induce connected subgraphs in *G*^*C *^and heavy subgraphs in *G*^*S*^. When weights can be both positive and negative (as is the case in our formulation), even the problem of finding a single heavy subgraph is NP-Hard (by a simple reduction from Max-Clique using a complete constraint graph). Hence, exact optimization is intractable, and we experimented with several heuristic algorithms for solving the problem. All the schemes share the following three phases: (1) detection of relatively small, high-scoring gene sets, or *seeds*, (2) seed improvement, and (3) significance-based filtering.

#### Identifying seeds

We tested three different methods for generating high scoring seeds. In all the methods a large set of non-overlapping potential seeds is first generated, and only seeds passing a certain score threshold are passed to the next phase.

##### Best-neighbors

In this method, high scoring seeds of a predefined size *k *are constructed. The nodes of the graph are ranked based on their total incident edge weights in *G*^*S *^(their *weighted degree*). The algorithm repeatedly creates a seed and removes its nodes from the graph. The seed generating step picks the highest ranking node *v*, and selects a set of *k *- 1 neighbors of *v *in *G*^*S *^that maximize the seed score. The optimal neighbor set can be found through exhaustive enumeration (enumeration is needed since the score for different neighbor sets depends also on the weights of the edges between them). When enumeration is computationally prohibitive, a heuristic that picks nodes with the highest weighted degree within the immediate neighborhood of *v *is utilized. Specifically, let *N*_*v *_be the set of all the immediate neighbors of *v*. For *i *∈ *N*_*v *_define wiv=∑vj∈Nvwij
 MathType@MTEF@5@5@+=feaafiart1ev1aaatCvAUfKttLearuWrP9MDH5MBPbIqV92AaeXatLxBI9gBaebbnrfifHhDYfgasaacH8akY=wiFfYdH8Gipec8Eeeu0xXdbba9frFj0=OqFfea0dXdd9vqai=hGuQ8kuc9pgc9s8qqaq=dirpe0xb9q8qiLsFr0=vr0=vr0dc8meaabaqaciaacaGaaeqabaqabeGadaaakeaacqWG3bWDdaqhaaWcbaGaemyAaKgabaGaemODayhaaOGaeyypa0ZaaabeaeaacqWG3bWDdaWgaaWcbaGaemyAaKMaemOAaOgabeaaaeaacqWG2bGDdaWgaaadbaGaemOAaOgabeaaliabgIGiolabd6eaonaaBaaameaacqWG2bGDaeqaaaWcbeqdcqGHris5aaaa@3FC3@. The heuristic selects *k *- 1 nodes with the highest *w*^*v *^values.

##### All-neighbors

This method is similar to Best-Neighbors, but instead of selecting *k *- 1 neighbors for a potential seed, in this version, all the neighbors of *v *with a non-negative edge score (including neighboring back nodes with zero score) enter the seed.

##### Heaviest-subnet

This method is inspired by Charikar's 2-approximation algorithm for the densest subgraph problem [46]. An *articulation node *in a connected graph is one whose removal disconnects the graph. The following algorithm is executed independently on each connected component in the constraint graph. The algorithm works in a "destructive" fashion: starting from the original constraint graph, nodes are removed from the graph one at a time until none remain. The next node to be removed is one with the smallest weighted degree in the current similarity graph that is not an articulation node in the current constraint graph. It is easy to see that such a node always exists. After each node removal, the overall score of the remaining graph is recorded. After all nodes are removed, the highest-scoring (possibly size-constrained) subgraph that was encountered is selected as the seed. That subgraph is then removed from the graph and the next seed is sought.

#### Seed optimization

**Figure 6:**
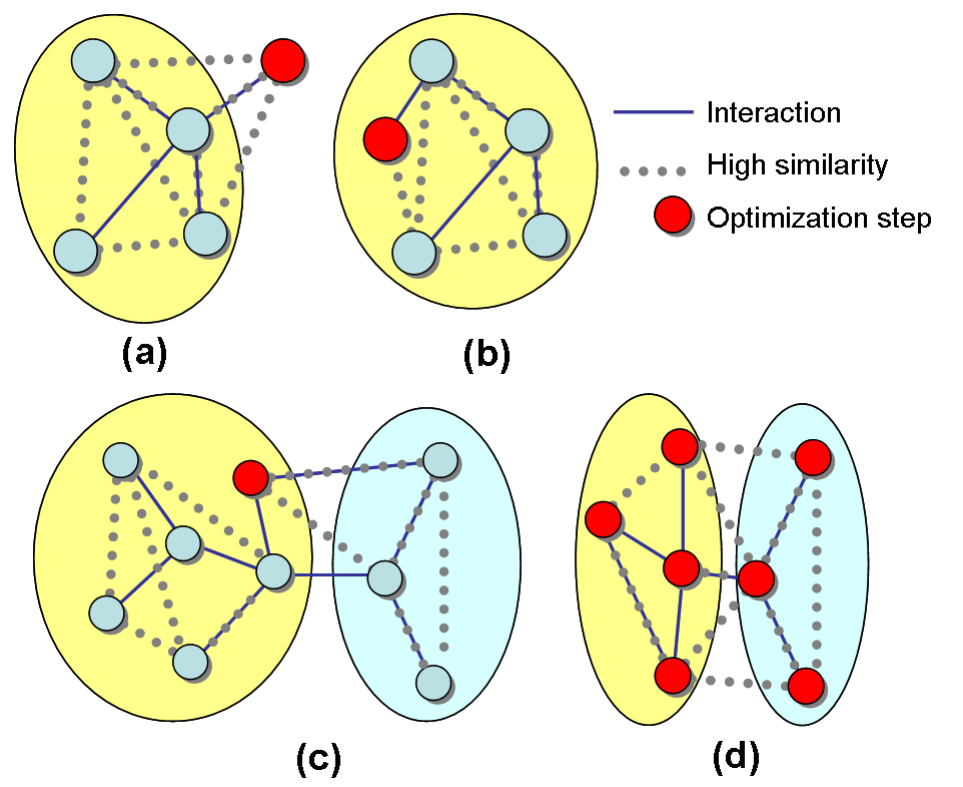
**Toy examples of the moves performed by the optimization algorithm**. (a) Node addition; (b) Node removal; (c) Assignment change; (d) JACS merge. In each case the affected nodes are in red (black).

Once a set of high-scoring seeds is established, a greedy algorithm aims to optimize all the seeds simultaneously. In our tests, this strategy worked better than optimizing each seed separately, as it produced more diverse JACSs. The algorithm keeps a set of disjoint subnetworks at every iteration and considers the following moves (Figure 6):

##### Node addition

Addition of an unassigned node to an existing JACS.

##### Node removal

Removal of a node from a JACS.

##### Assignment change

Exchange of a node between JACSs.

##### JACS merge

A new JACS is formed by taking the union of the nodes in two existing JACSs. This step is particularly beneficial when the original seeds are relatively small.

At every step a move is selected only if (1) it improves the overall score of the solution, i.e., the sum of the weights of all the JACSs and (2) the move maintains the connectivity of the JACSs. If no such step exists, a "cleanup" procedure iteratively removes from every JACS non-articulation back nodes that are not found on any simple path between front nodes. If the clean-up step does not remove any nodes, the optimization halts. Note that the algorithm is guaranteed to converge, as the global score is monotonically increasing. In addition, in order to obtain biologically meaningful JACSs, an upper bound on the size of a JACS can be employed throughout the optimization. If a JACS reaches this upper bound in the course of the optimization, any node added to it causes a removal of a low-scoring node, maintaining the JACS size. Note that this procedure can add only front nodes.

#### Filtering

After a collection of putative JACSs is obtained, it is filtered based on the significance of the JACS score. For that purpose, for every candidate JACS, an empirical p-value of its score is calculated using sampling randomly gene groups of the same size. Only candidate JACSs with p-value below a threshold *p *pass the filtering stage (*p *= 0.05 after Bonferroni correction was used). In a second step, to avoid possible bias in the score, we empirically test the JACS significance using only expression similarity scores. The same sampling procedure is performed using the average raw expression pairwise similarity values, and JACSs whose average similarity is not sufficiently high compared to the sampled sets of the same size are removed. An efficient computation of this step is done as suggested in [15].

### Implementation issues

For efficient implementation, several slight modifications were made to the algorithm described above:

#### Removal of non-contributing nodes

As in our framework only front nodes are used for JACS scoring, back nodes will be incorporated into the subnetwork only if they appear on some path between two front nodes. Thus, prior to algorithm execution we remove from *G*^*C *^all back nodes that are leaves (nodes with degree smaller than 2). The procedure is iterated until no such leaves remain in the graph. In practice, due to the nature of the protein interaction network used, this step significantly reduces the size of the network, without influencing the quality of the solution.

#### Similarity graph adjustment

When finding Heaviest-Subnet seeds, low edge density in the graph is crucial for efficiency. We therefore remove edges with low absolute weight from the graph, as their contribution to the overall JACS score is small. All the edges are used in the subsequent phases.

#### Finding heaviest-subnet seeds

Efficient implementation of this algorithm can be done using a data structure similar to the one developed for the dynamic connectivity problem [47]. This would take *O*(|*V*|log^4 ^|*V*|) time per seed. Instead, we used a simple algorithm for detection of articulation nodes in each iteration. Articulation nodes can be detected during a depth-first traversal of the graph, by calculating the "lowpoint" values of every node (cf. [48]).

This implementation required complexity of *O*(|*V*||*E*^*S*^|) time per seed. Since this time can be too long for very large graphs, we use a sampling approach when the component contains more than 1,500 nodes: a connected subgraph of a more modest size is randomly sampled (as described in [49]) and then used for seed finding. This sampling is repeated several times, with the highest scoring seed used for further optimization.

#### Implementation

**Figure 7:**
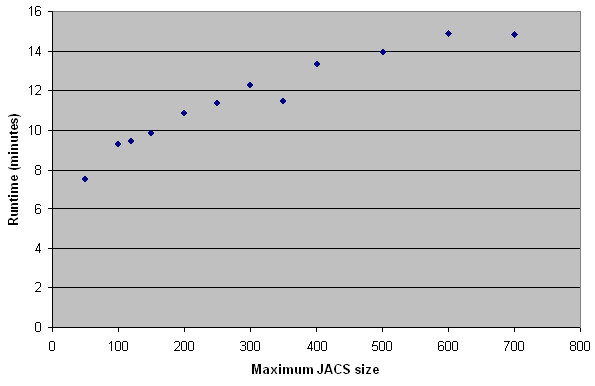
**Dependence of the running time on the size of the JACS**. The running time of MATISSE with different maximum JACS size parameters. The execution did not include the weight calculation step, as it is not dependent on the JACS size.

MATISSE was implemented as a Java stand-alone application. In addition to the algorithmic engine, it contains a visualization tool allowing flexible inspection of the obtained subnetworks and diverse post-process analyses. Running times are efficient enough to accommodate large interaction networks and gene expression datasets. For example, on a constraint graph of 4, 543 nodes and 1, 996 expression profiles, the processing took less than 15 minutes for All-Neighbors and Best-Neighbors methods and 78 minutes for Heaviest-Subnet, on a Pentium 4 3 GHz machine with 2 GB memory. About 10 – 20% of the time is needed to learn the parameters using EM, and this time is saved in all subsequent runs on the same data. The running time depends sublinearly on the bound on the maximum size of the JACS (Figure 7). The application will soon be available at [50].

### Simulation setup

Our simulations used the real connected network of 2,000 yeast proteins described in Results, and synthetic similarity values, generated as follows. First, a set of *m *disjoint connected subnetworks *P*_1_,..., *P*_*m *_of equal size *k *was randomly selected as in [49]. Then, from each subnetwork a subset of size *k*·*p*_*f *_was randomly selected to be included in *V*_*sim *_(front nodes). The resulting *V*_*sim *_was expanded by additional randomly selected nodes, to contain *n*_*sim *_nodes in total. Similarity values were generated as in [27] using two Gaussian distributions - *N*_*m *_with parameters *μ*_*m*_, *σ*_*m *_for similarity between mates and *N*_*n *_with parameters *μ*_*n*_, *σ*_*n *_for all other pairs.

Similarity values were determined independently for each node pair, as follows: If the two nodes reside in the same JACS, the value was drawn from *N*_*m *_with probability *β*_*m *_and from *N*_*n *_with probability 1 - *β*_*m*_. Otherwise, the value was drawn from *N*_*m *_with probability *p*_*m*_.

The default values for the simulations were set to *n*_*sim *_= 1, 000 (out of |*V*| = 2, 000);

*m *= 6;*k *= 100;*p*_*f *_= 0.7;*μ*_*m *_= 0.5;*μ*_*n *_= 0;*σ*_*m *_= *σ*_*n *_= 0.3;*β*_*m *_= 0.95;*p*_*m *_= 0.01.

### Evaluating performance

The success of an algorithm in recovering the planted components was measured using the Jaccard coefficient [51]. It is defined as n11n11+n10+n01
 MathType@MTEF@5@5@+=feaafiart1ev1aaatCvAUfKttLearuWrP9MDH5MBPbIqV92AaeXatLxBI9gBaebbnrfifHhDYfgasaacH8akY=wiFfYdH8Gipec8Eeeu0xXdbba9frFj0=OqFfea0dXdd9vqai=hGuQ8kuc9pgc9s8qqaq=dirpe0xb9q8qiLsFr0=vr0=vr0dc8meaabaqaciaacaGaaeqabaqabeGadaaakeaadaWcaaqaaiabd6gaUnaaBaaaleaacqaIXaqmcqaIXaqmaeqaaaGcbaGaemOBa42aaSbaaSqaaiabigdaXiabigdaXaqabaGccqGHRaWkcqWGUbGBdaWgaaWcbaGaeGymaeJaeGimaadabeaakiabgUcaRiabd6gaUnaaBaaaleaacqaIWaamcqaIXaqmaeqaaaaaaaa@3C5E@, where *n*_11 _is the number of node pairs included both in the same planted component and in the same JACS, *n*_10 _is the number of pairs included in the same planted component but not in the same JACS, and *n*_01 _is the number of pairs in the same JACS but not in the same planted component. Hence, a perfect fit of the two solutions would get a score of 1, and lower scores indicate reduced fit.

### Parameter estimation

To obtain meaningful results, a good assessment of the parameters of the probabilistic model is prerequisite. We tested different schemes for assessing *P*(*R*_*i*_), and selected the following scheme. We ranked the genes based on the variation observed across their expression patterns and then applied a logistic function to the normalized ranks to obtain: *P*(*R*_*i*_) = *α *+ (1 - *α*)11+e−β(xi−γ)
 MathType@MTEF@5@5@+=feaafiart1ev1aaatCvAUfKttLearuWrP9MDH5MBPbIqV92AaeXatLxBI9gBaebbnrfifHhDYfgasaacH8akY=wiFfYdH8Gipec8Eeeu0xXdbba9frFj0=OqFfea0dXdd9vqai=hGuQ8kuc9pgc9s8qqaq=dirpe0xb9q8qiLsFr0=vr0=vr0dc8meaabaqaciaacaGaaeqabaqabeGadaaakeaadaWcaaqaaiabigdaXaqaaiabigdaXiabgUcaRiabdwgaLnaaCaaaleqabaGaeyOeI0ccciGae8NSdiMaeiikaGIaemiEaG3aaSbaaWqaaiabdMgaPbqabaWccqGHsislcqWFZoWzcqGGPaqkaaaaaaaa@3AE0@, where *x*_*i *_is the normalized rank of gene *i*. The logistic parameters were empirically set to *α *= 0.6, *β *= 24 and *γ *= 0.25. To evaluate the effect of the specific form of the prior on the results, we reran the JACS finding algorithms with different logistic parameter settings (*α *= 0.4..0.8, *β *= 1..24, *γ *= 0.2..0.7). The average expression homogeneity and the average functional homogeneity of the produced JACSs (computed as described in [1]) of the JACSs did not change by more than 6%.

We adjusted the standard EM algorithm used for learning a mixture of Gaussians (cf. [52]) in order to estimate *μ*_*m*_, *σ*_*n*_, *μ*_*n*_, *σ*_*n *_and *p*_*m*_. A detailed description of the EM algorithm can be found at our website ([50]). The produced JACSs were constrained to the size range of 5–120 and *β*_*m *_was set to 0.9. We verified that the reported results are robust to changes in the value of *β*_*m *_by varying it between 0.75 and 0.99 and analyzing the obtained solutions. We found that both the average expression homogeneity and the average functional homogeneity did not change by more than 3% across this parameter range.

### Comparison of the heuristics

**Figure 8:**
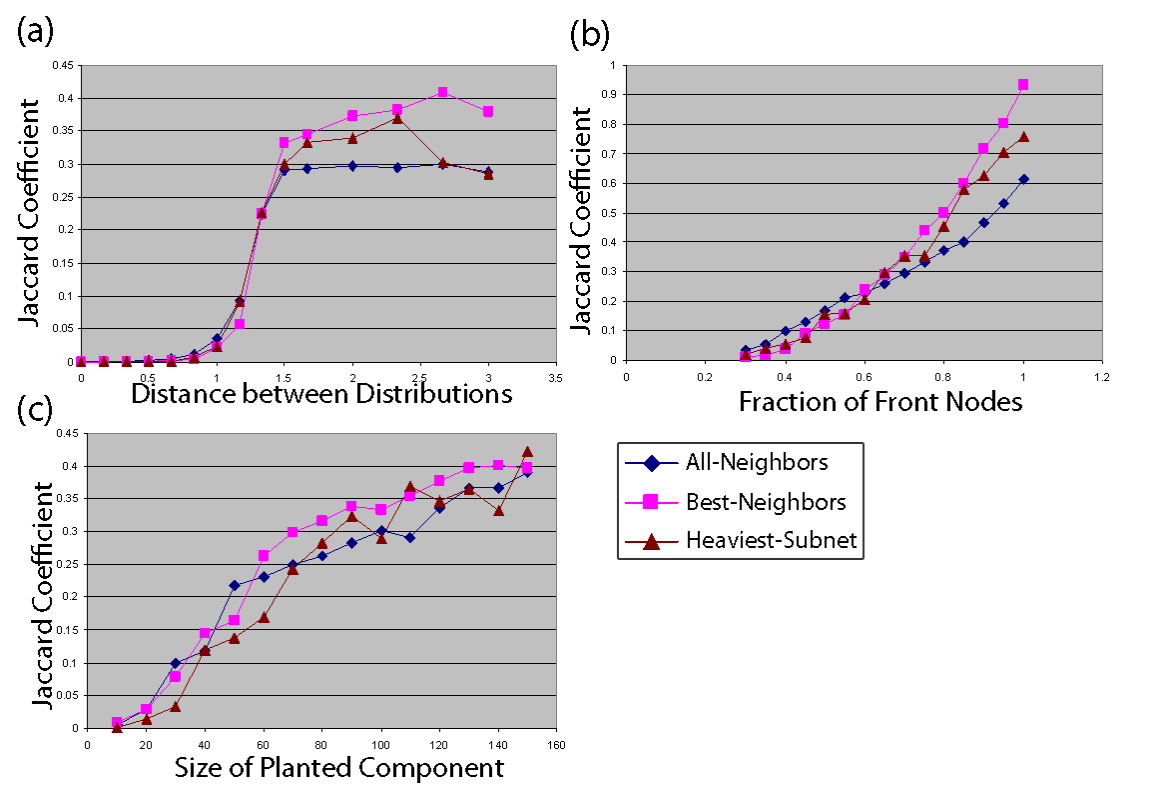
**Performance of the three proposed heuristics on simulated data**. See Figure 2 for further details.

We evaluated the three proposed heuristics both in our simulation setting and on the osmotic shock response in *S. cerevisiae*. The results of the comparison on simulation data are presented in Figure 8. Overall, as can be seen in Figure 8, all three MATISSE variants show similar performance. All the methods exhibit poor performance in detection of small planted components (*k *< 50). Best-Neighbors seems to be the preferred method on the simulated data. Best-Neighbors and All-Neighbors is that Best-Neighbors does not incorporate back nodes at all, while All-Neighbors may include some. As we shall show below, using back nodes is in fact advantageous in real biological data. The performance of the Heaviest-Subnet seeding is highly variable, probably due to its relatively significant dependency on the structure of the similarity graph.

**Figure 9:**
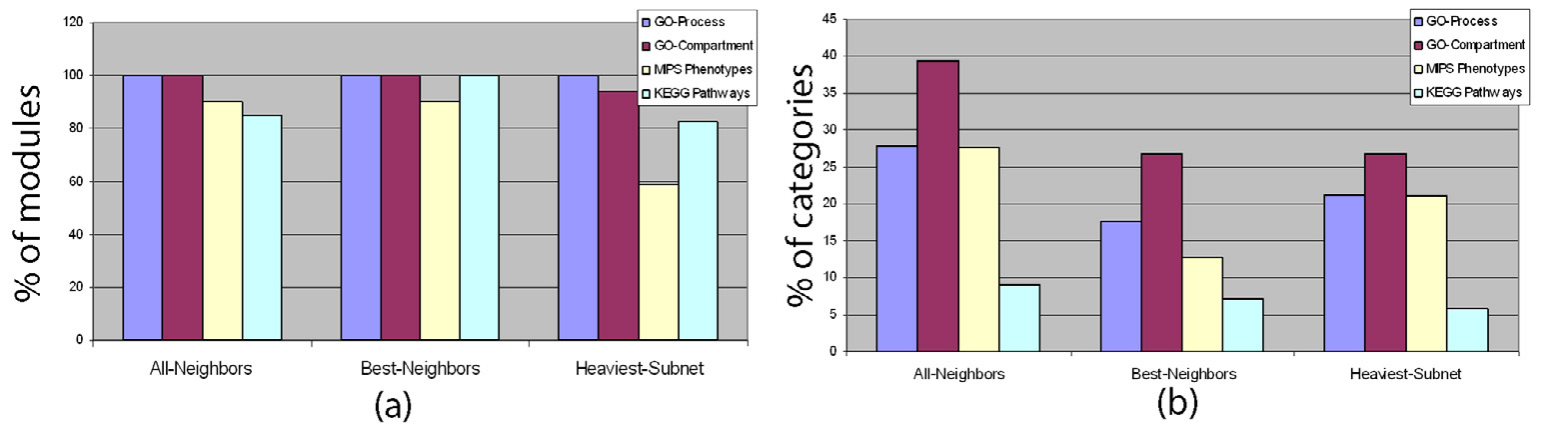
**Performance of the three proposed heuristic in terms of annotation enrichment**. See Figure 3 for further details.

The results of the comparison on simulation data are presented in Figure 9. The Best-Neighbors variant performs slightly better than All-Neighbors in terms of the fraction of enriched modules, but All-Neighbors performs significantly better in terms of category coverage, due to its inclusion of back nodes. We therefore carried out all subsequent analysis using the modules produced with the All-Neighbors variant.

### Functional enrichment analysis

We used the TANGO algorithm [31] for finding GO terms enriched in the JACSs. The algorithm considers all levels of GO and corrects p-values for multiple testing and for category dependency using resampling. Briefly, TANGO repeatedly selects random sets of genes to compute an empirical distribution of maximum p-values for functional enrichment obtained across a random sample of sets that maintain the same size characteristics of the ones analyzed. TANGO uses this empirical distribution to determine thresholds for significant enrichment on the true clusters. The algorithm filters out redundant categories by performing conditional enrichment tests that ensure that all the reported enriched categories are statistically significant even after taking into account the enrichment of their ancestor and children nodes in the tree.

### Extraction of subnetwork hubs

Given a JACS *J*, *v *∈ *J *was called a *hub *if it satisfied three requirements: (a) the degree of *v *within the subnetwork *J *exceeds 7; (b) the degree of *v *in *J *is among the five highest in *J*; (c) the degree of *v *in *J *is significantly high given its degree in the whole network (*p *< 0.05 using hypergeometric distribution). Note that back nodes can also be hubs.

## Authors' contributions

IU and RS designed the study. IU developed MATISSE and performed the statistical analysis. IU and RS wrote the manuscript. Both authors read and approved the final manuscript.

## Supplementary Material

Additional File 1 **JACSs identified by MATISSE**. Images of the subnetworks identified by MATISSE in the osmotic shock response and the cell cycle datasets. Click here for file

## Figures and Tables

**Table 1:** Performance of the different module finding algorithms on the S. cerevisiae osmotic shock data

Solution	No. of modules	Total nodes	Average size	Expression homogeneity	Clustering coefficient	Edge density	No. of connected components
MATISSE	20	2107	105.35	0.361	0.073	0.035	1.00
Co-clustering	19	1991	104.79	0.354	0.035	0.010	89.67
CLICK	20	1988	99.40	0.438	0.030	0.011	77.61
Random connected	20	2107	105.35	0.063	0.050	0.036	1.00
Random	20	2105	105.35	0.033	0.004	0.003	89.78

Numbers in columns 4–8 are averages over all the modules in each solution.

**Table 2:** Functionally enriched modules found in the yeast osmotic shock data

JACS	Size	Front	Enriched GO terms	p-value	TFs	p-value
1	120	119	processing of 20S pre-rRNA	< 0.001	Fhl1	4.82·10^-16^
			rRNA processing	< 0.001	Rap1	2.89·10^-11^
			35S primary transcript processing	< 0.001	Sfp1	2.98·10^-8^
			ribosomal large subunit assembly and maintenance	0.019		
			rRNA modification	< 0.001		
			ribosome biogenesis	0.029		
2	120	118	translational elongation	< 0.001	Fhl1	1.03·10^-5^
3	120	118	processing of 20S pre-rRNA	< 0.001		
			rRNA processing	0.030		
			35S primary transcript processing	0.011		
			ribosomal large subunit assembly and maintenance	0.019		
			ribosomal large subunit biogenesis	< 0.001		
5	120	112	signal transduction during filamentous growth	0.010	Ste12	5.41·10^-13^
			conjugation with cellular fusion	< 0.001	Dig1	5.41·10^-13^
6	120	99	transcription from RNA polymerase III promoter	< 0.001		
			transcription from RNA polymerase I promoter	0.006		
7	120	107	ergosterol biosynthesis	< 0.001		
			hexose transport	0.019		
8	114	85	chromatin remodeling	0.050		
11	120	114	pseudohyphal growth	0.010	Msn2	3.17·10^-4^
			response to stress	< 0.001	Msn4	1.82·10^-12^
14	120	102	ubiquitin-dependent protein catabolism	0.047		
15	120	96	nuclear mRNA splicing, via spliceosome	< 0.001		
16	89	61	ubiquitin-dependent protein catabolism	< 0.001	Rpn4	6.44·10^-6^
17	120	109	response to stress	< 0.001	Msn4	1.74·10^-3^
			mitochondrial electron transport	< 0.001		
18	87	59	nuclear mRNA splicing, via spliceosome	0.012		
20	46	35	pyridoxine metabolism	0.045		

The GO p-value was adjusted for multiple testing using the TANGO algorithm (see Methods). Enriched TF binding site motifs were detected using the PRIMA algorithm [35]. TF p-values were Bonferroni corrected for multiple testing.
