# Supplementary material for: Identification of functional modules using network topology and high-throughput data
Source: BMC Syst Biol. 2007 Jan 26;1:8. doi: 10.1186/1752-0509-1-8 (PMC1839897; doi:10.1186/1752-0509-1-8)
Supplement: Additional File 1 — JACSs identified by MATISSE. Images of the subnetworks identified by MATISSE in the osmotic shock response and the cell cycle datasets. [file 1752-0509-1-8-S1.pdf]

1 Osmotic shock response in S. cerevisiae

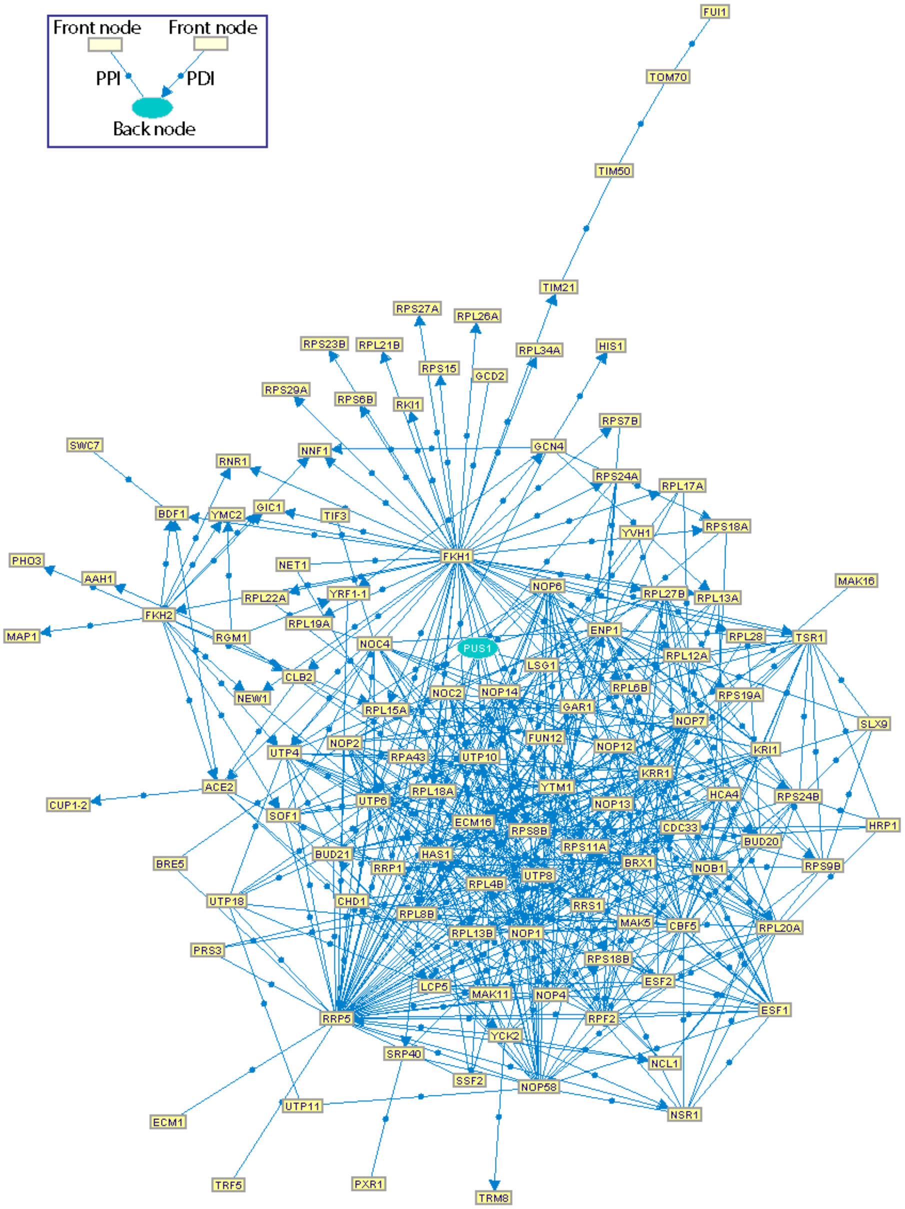

Figure 1: Osmotic shock response - JACS 1.

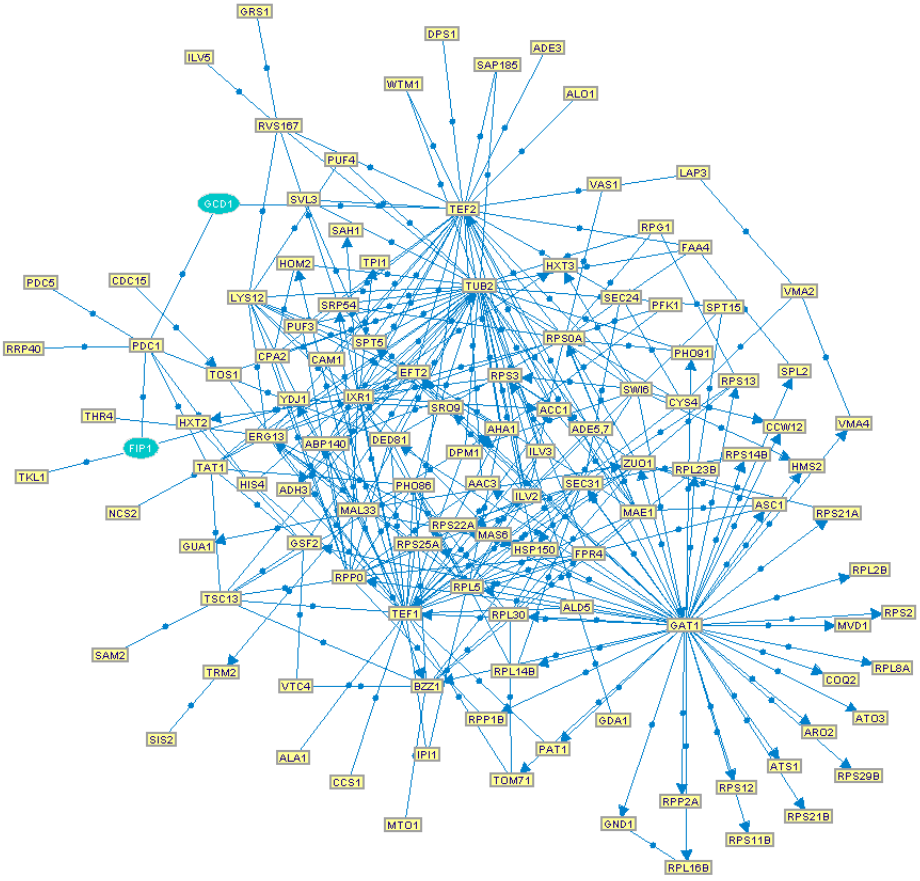

Figure 2: Osmotic shock response - JACS 2.

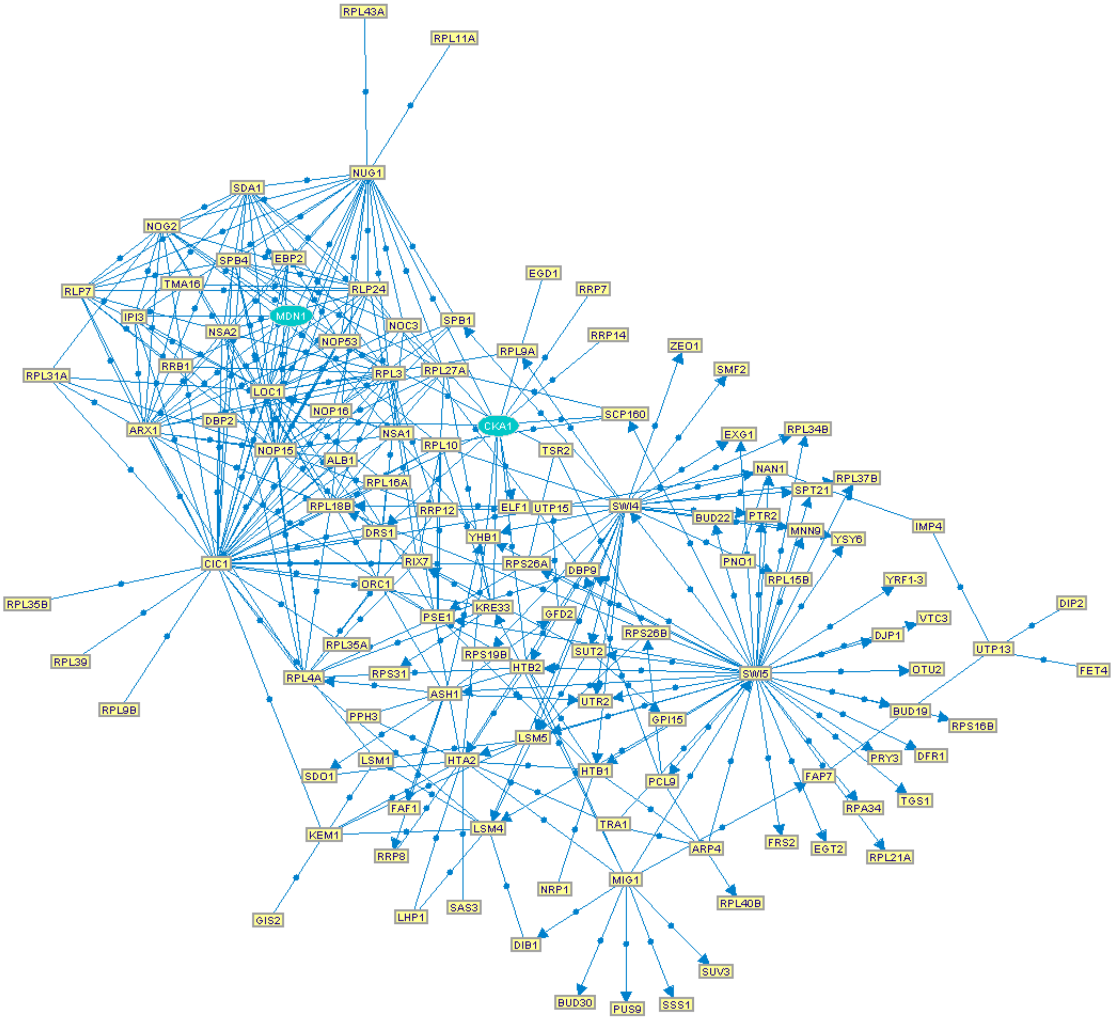

Figure 3: Osmotic shock response - JACS 3.

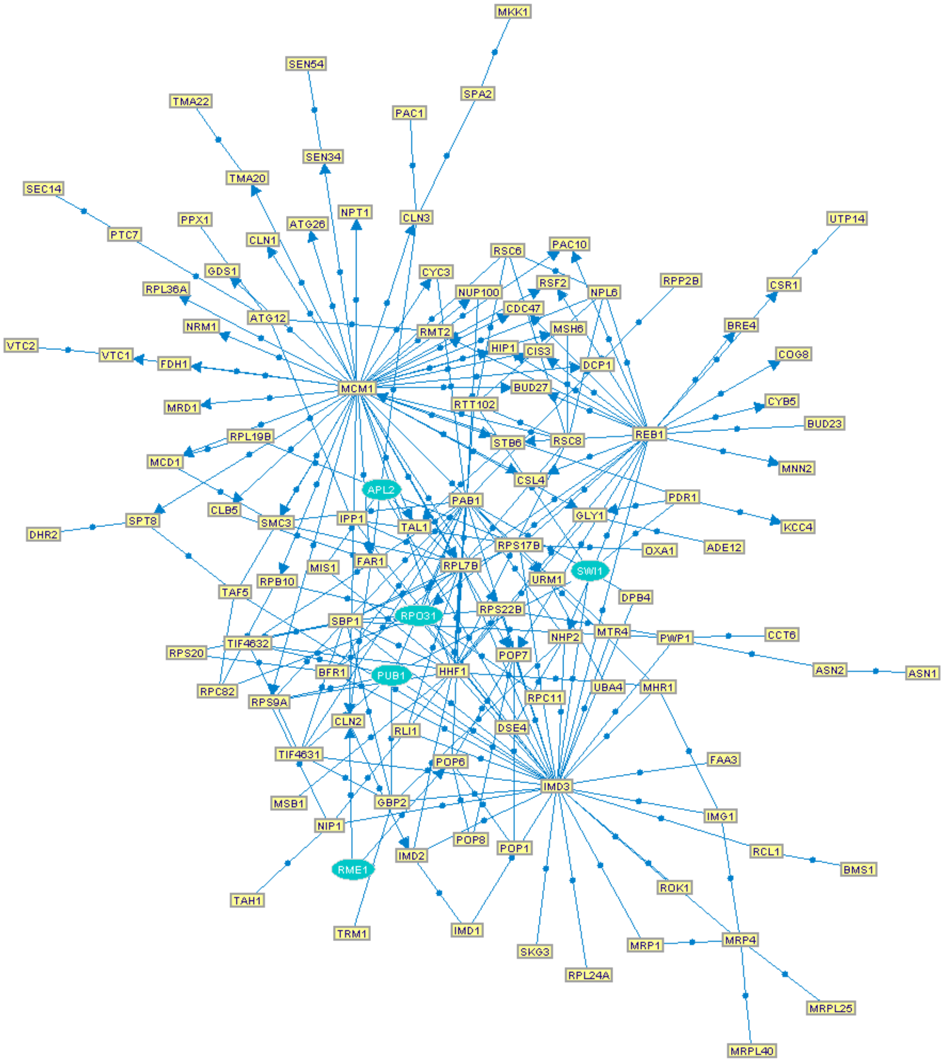

Figure 4: Osmotic shock response - JACS 4.

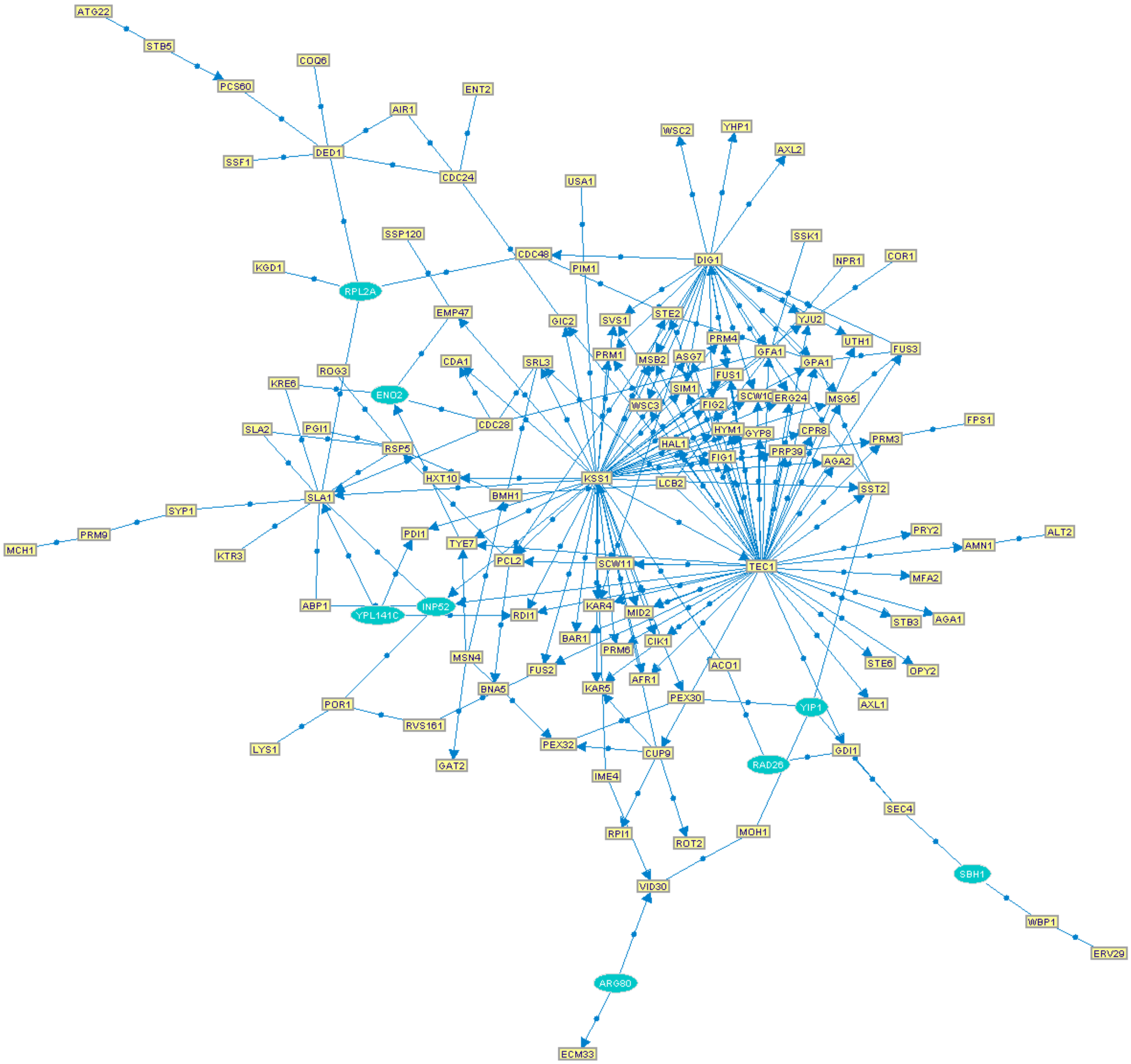

Figure 5: Osmotic shock response - JACS 5.

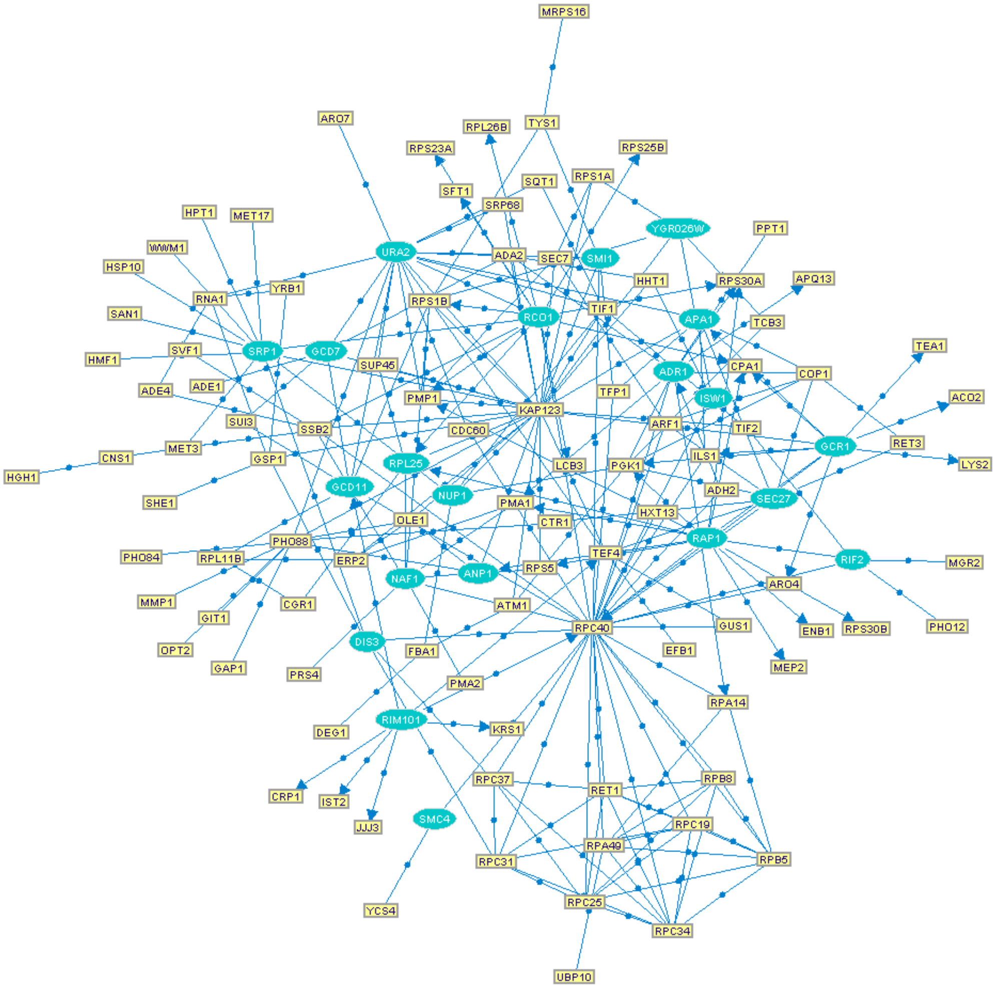

Figure 6: Osmotic shock response - JACS 6.

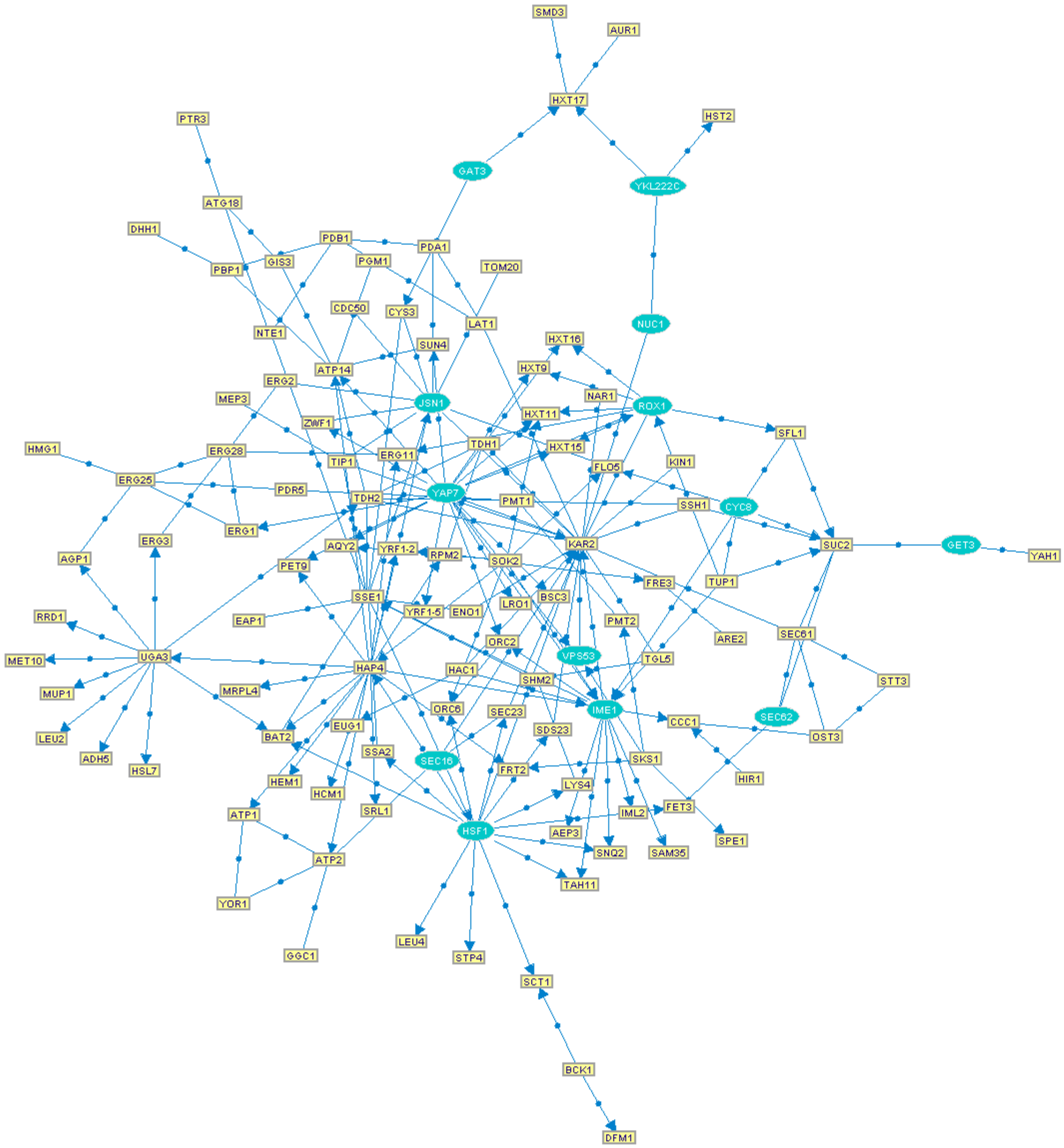

Figure 7: Osmotic shock response - JACS 7.

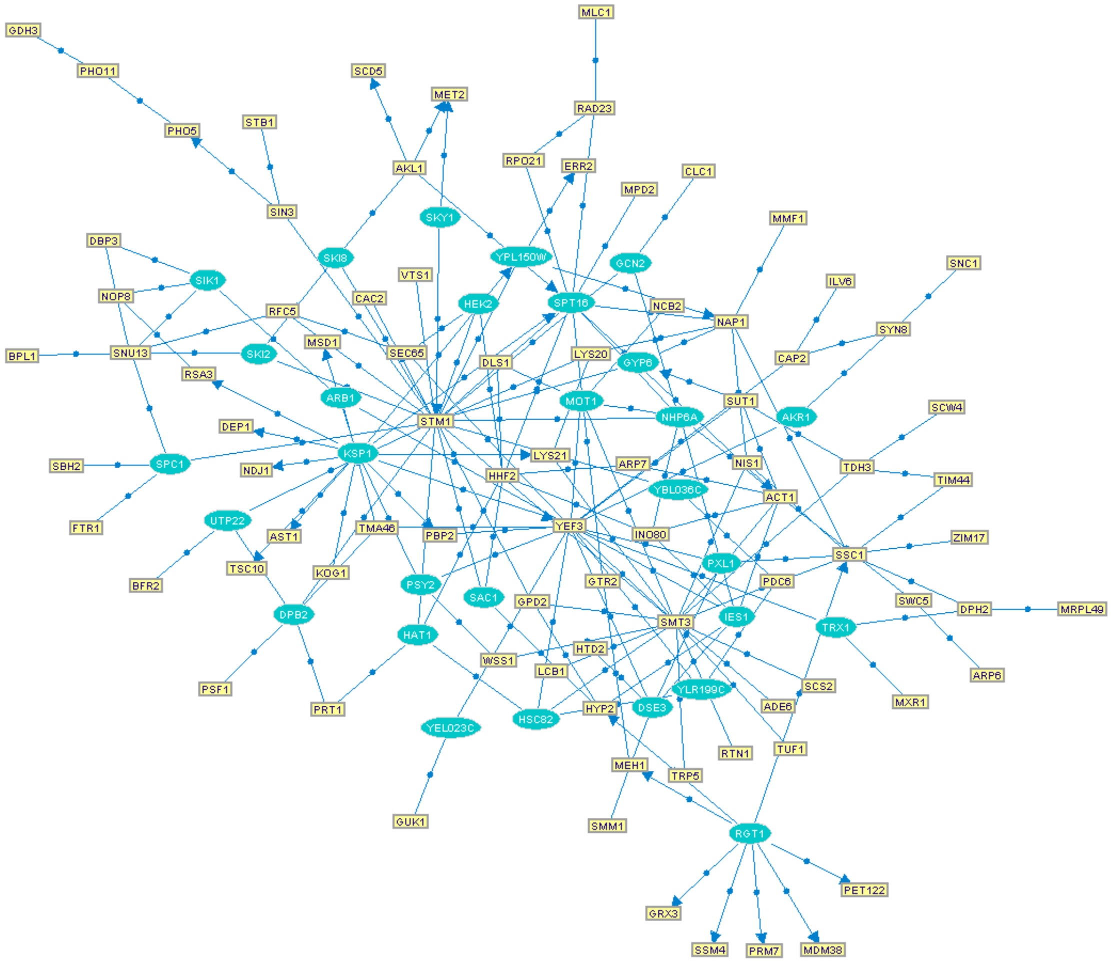

Figure 8: Osmotic shock response - JACS 8.

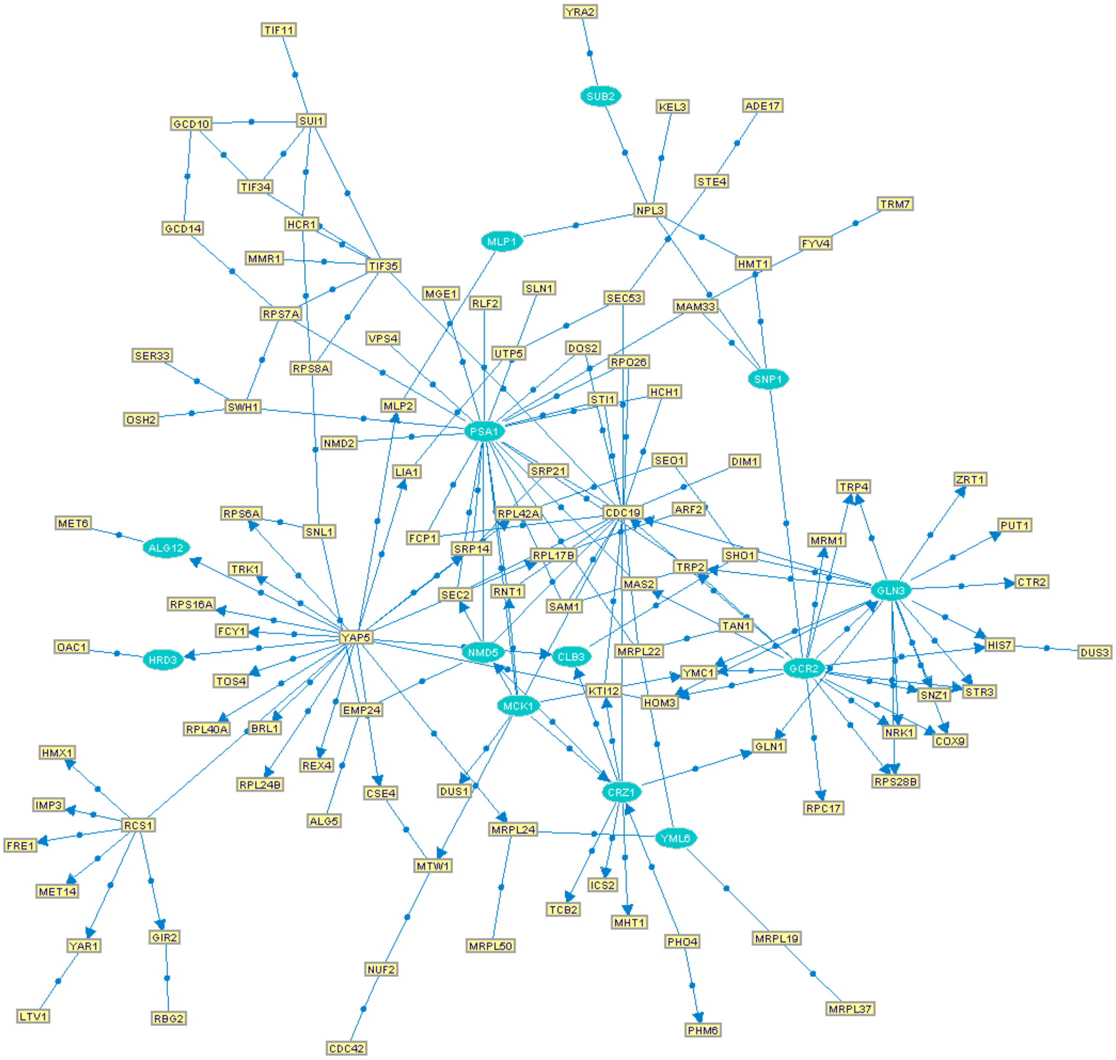

Figure 9: Osmotic shock response - JACS 9.

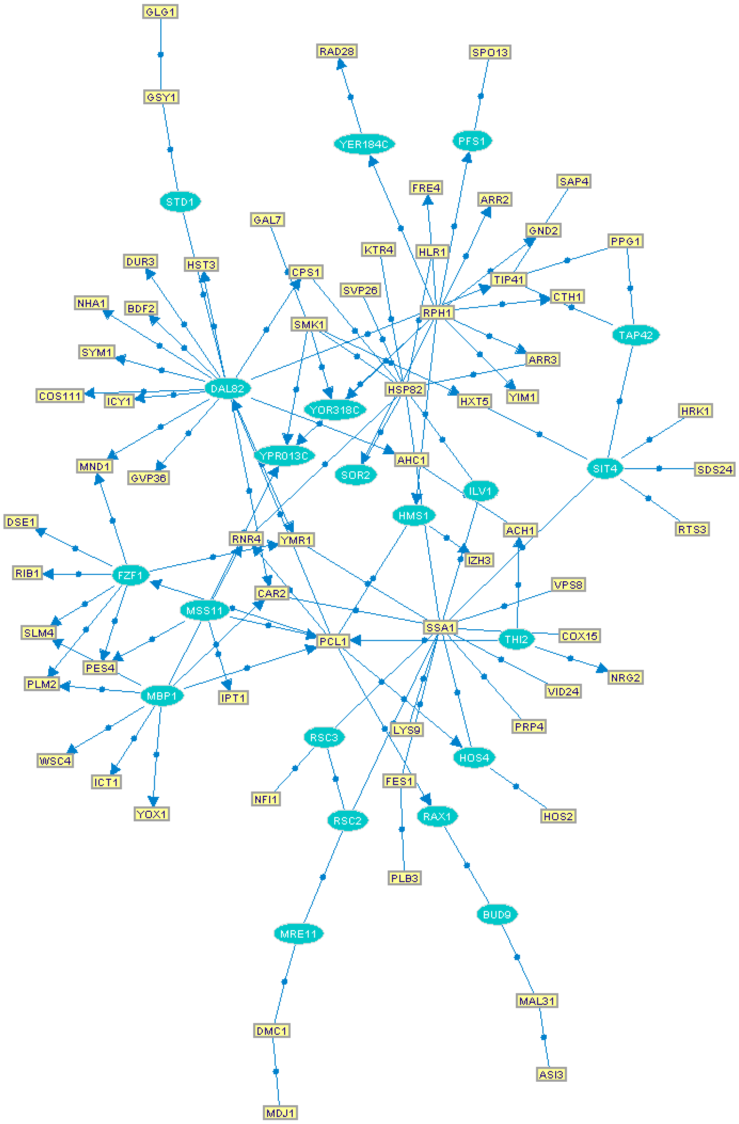

Figure 10: Osmotic shock response - JACS 10.

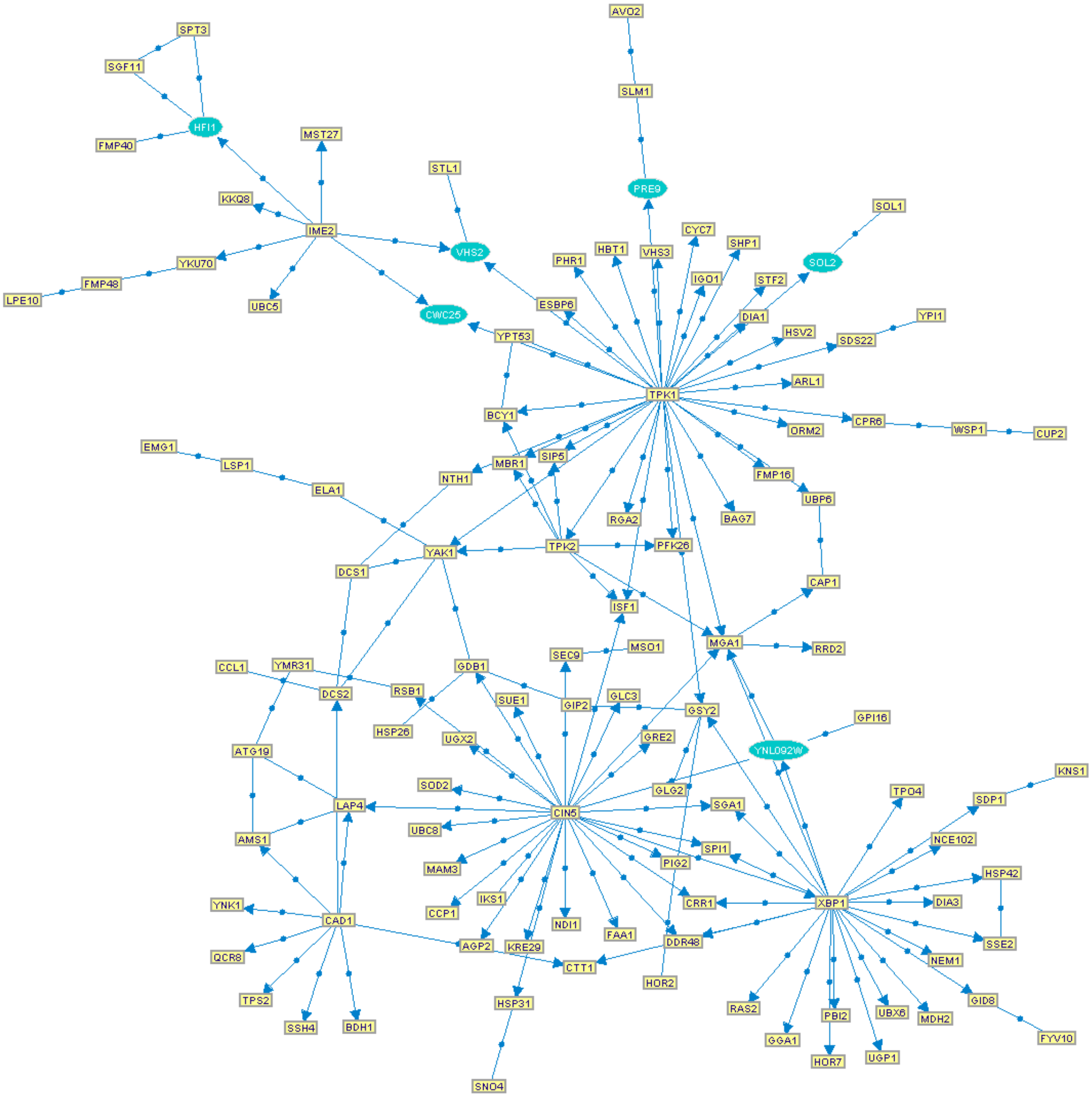

Figure 11: Osmotic shock response - JACS 11.

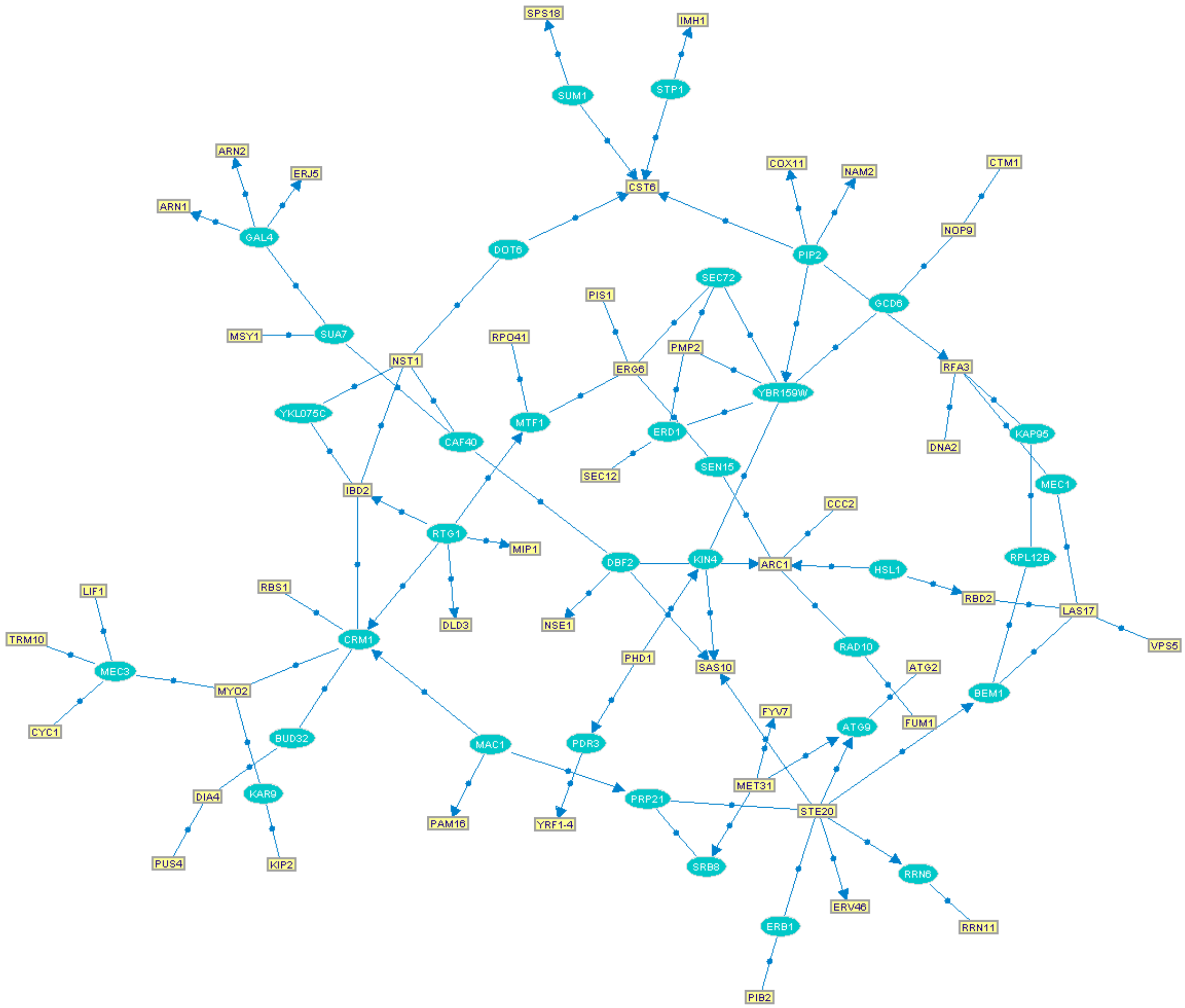

Figure 12: Osmotic shock response - JACS 12.

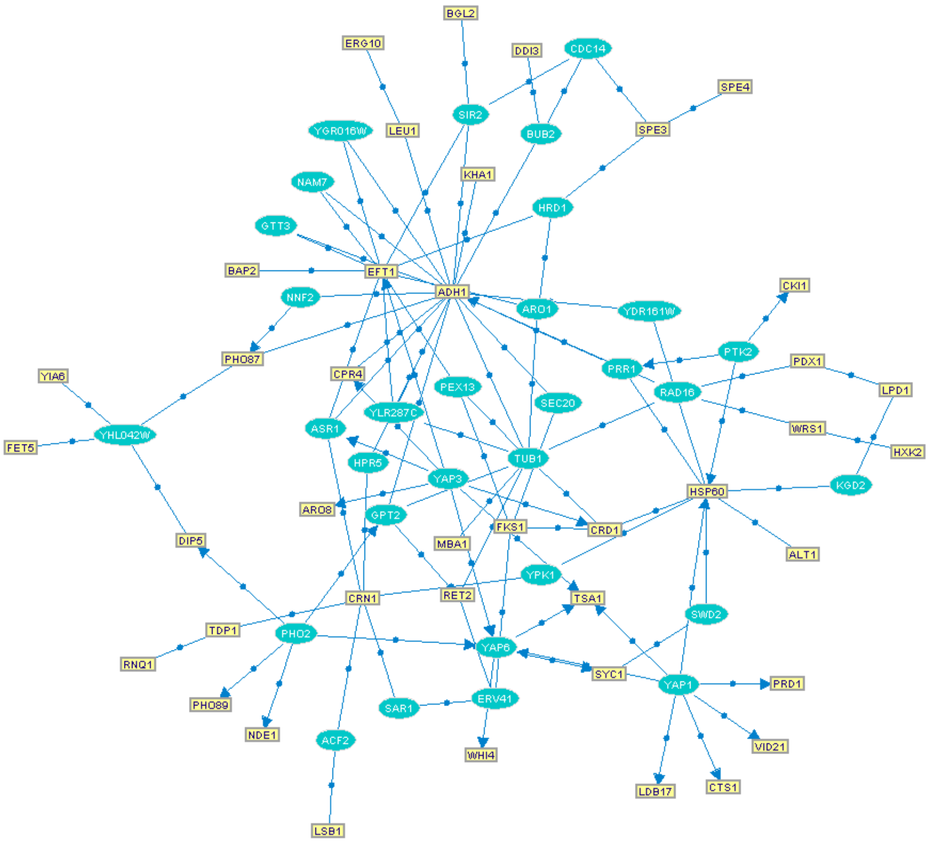

Figure 13: Osmotic shock response - JACS 13.

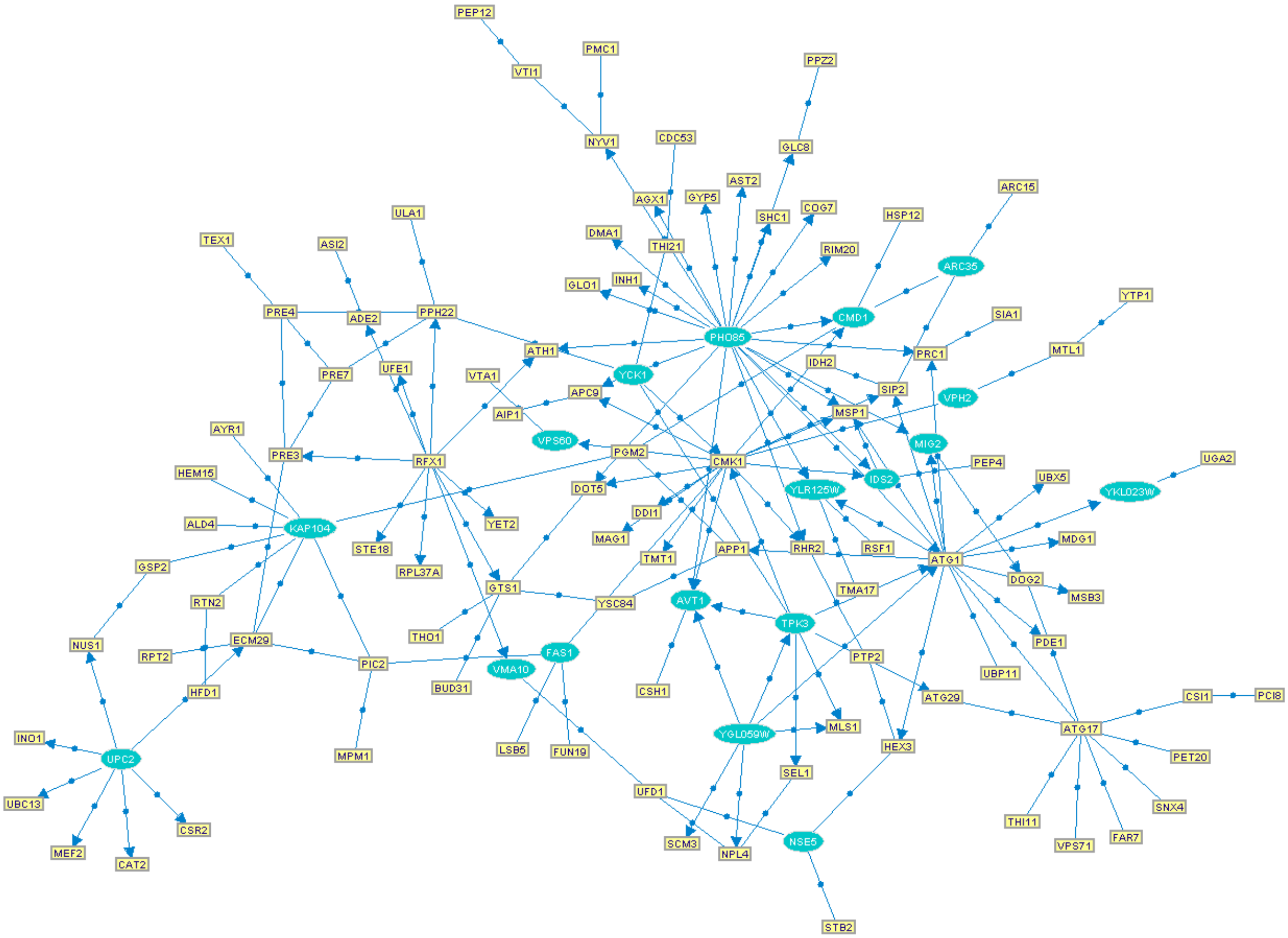

Figure 14: Osmotic shock response - JACS 14.

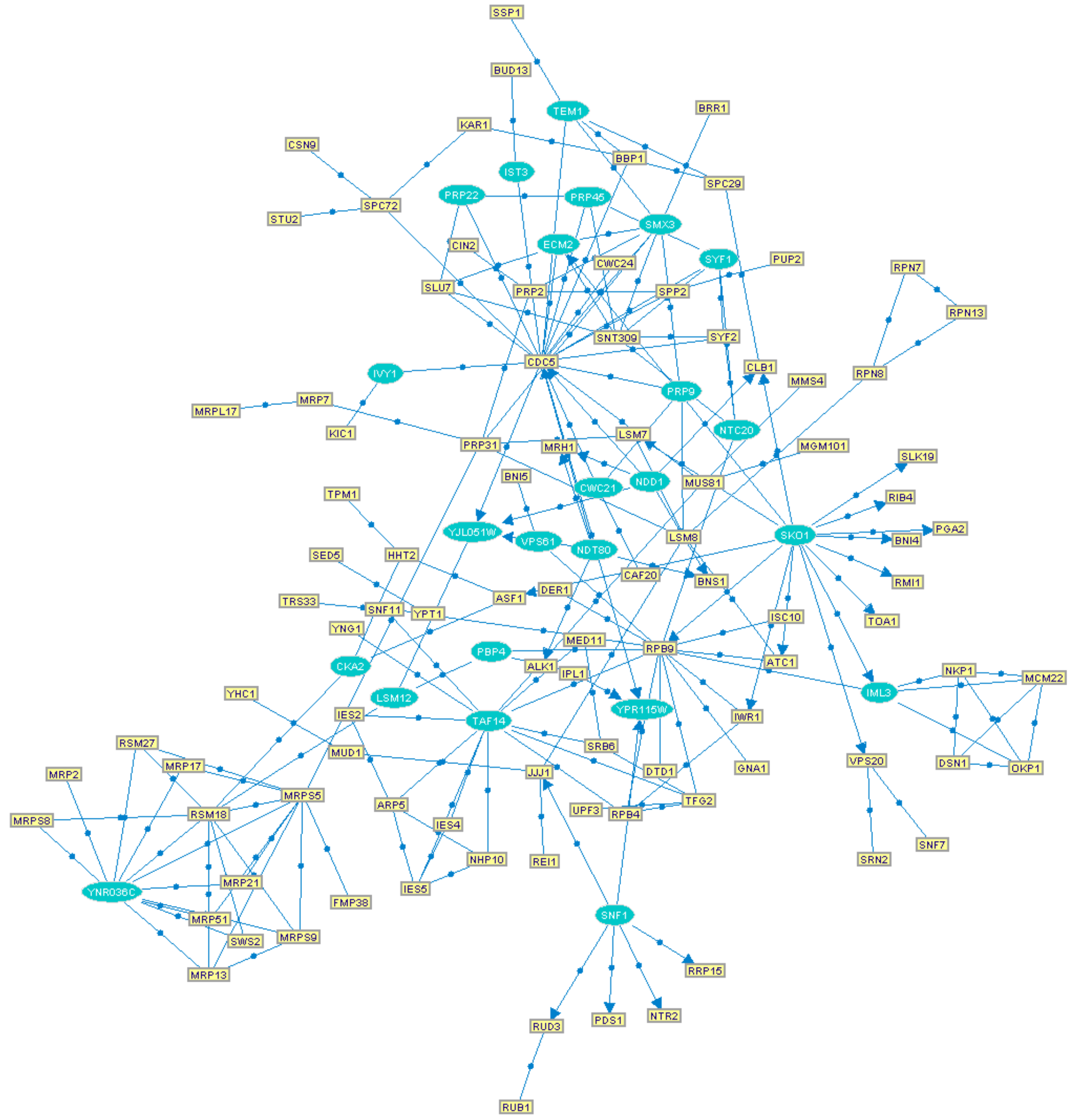

Figure 15: Osmotic shock response - JACS 15.

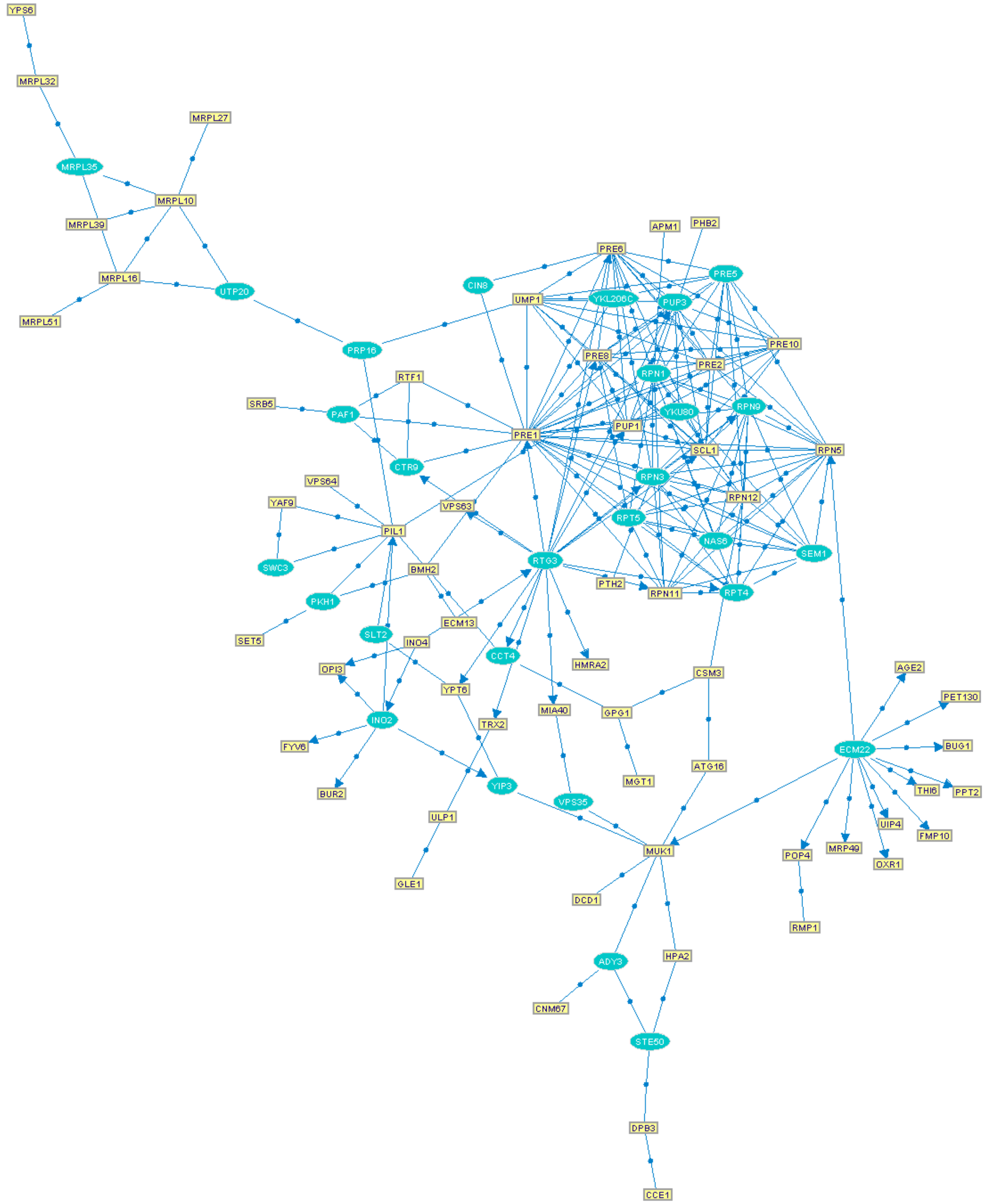

Figure 16: Osmotic shock response - JACS 16.

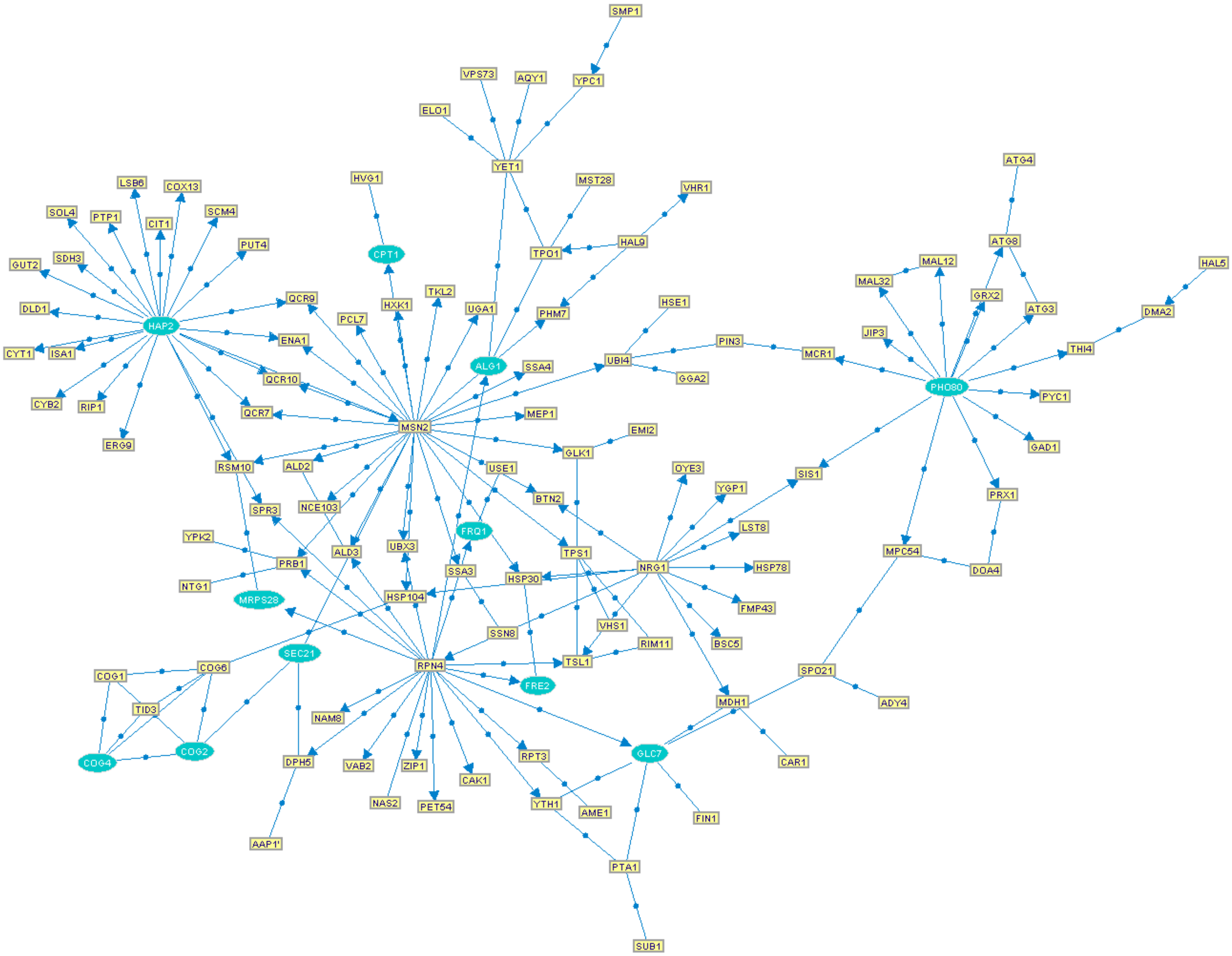

Figure 17: Osmotic shock response - JACS 17.

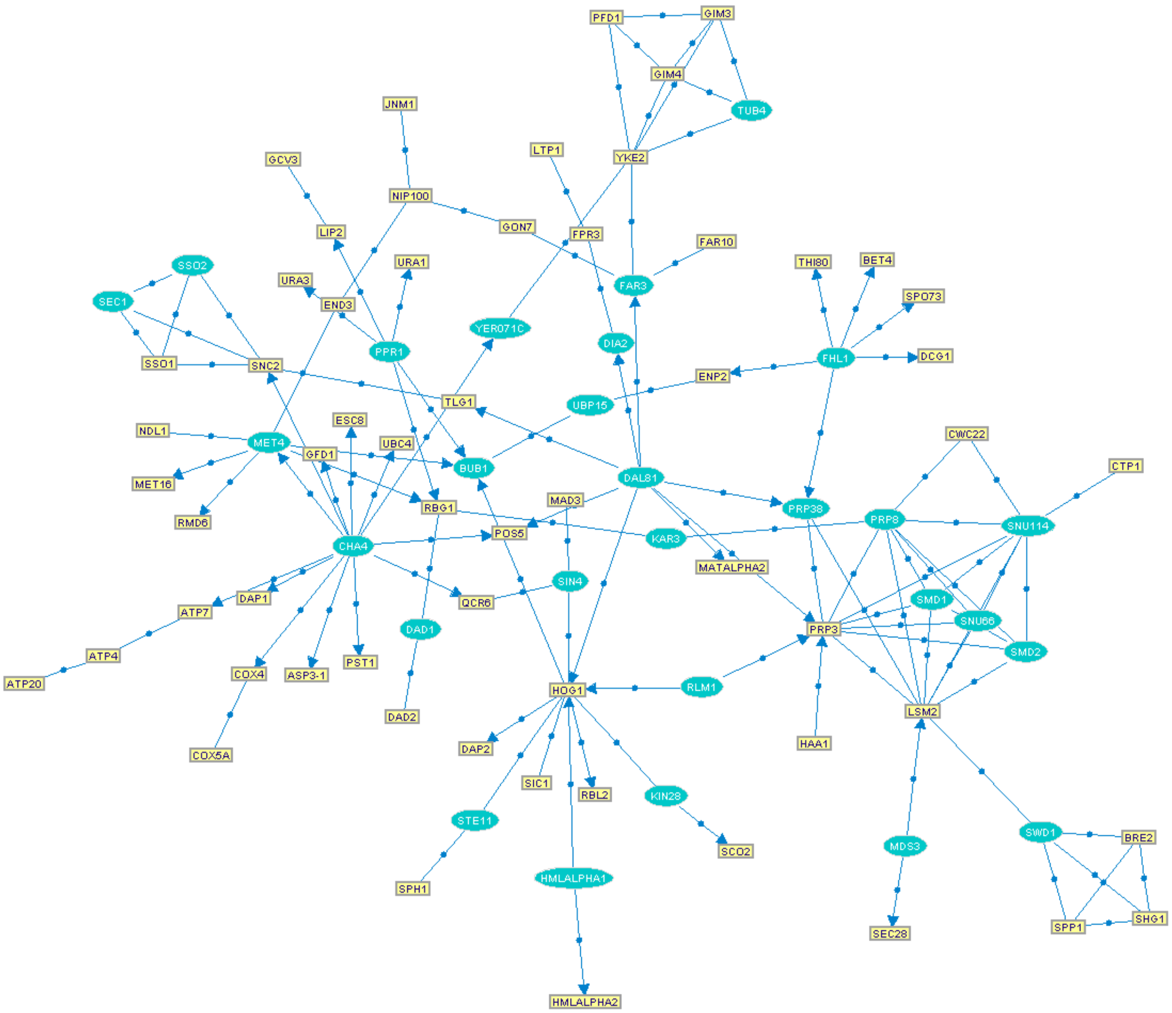

Figure 18: Osmotic shock response - JACS 18.

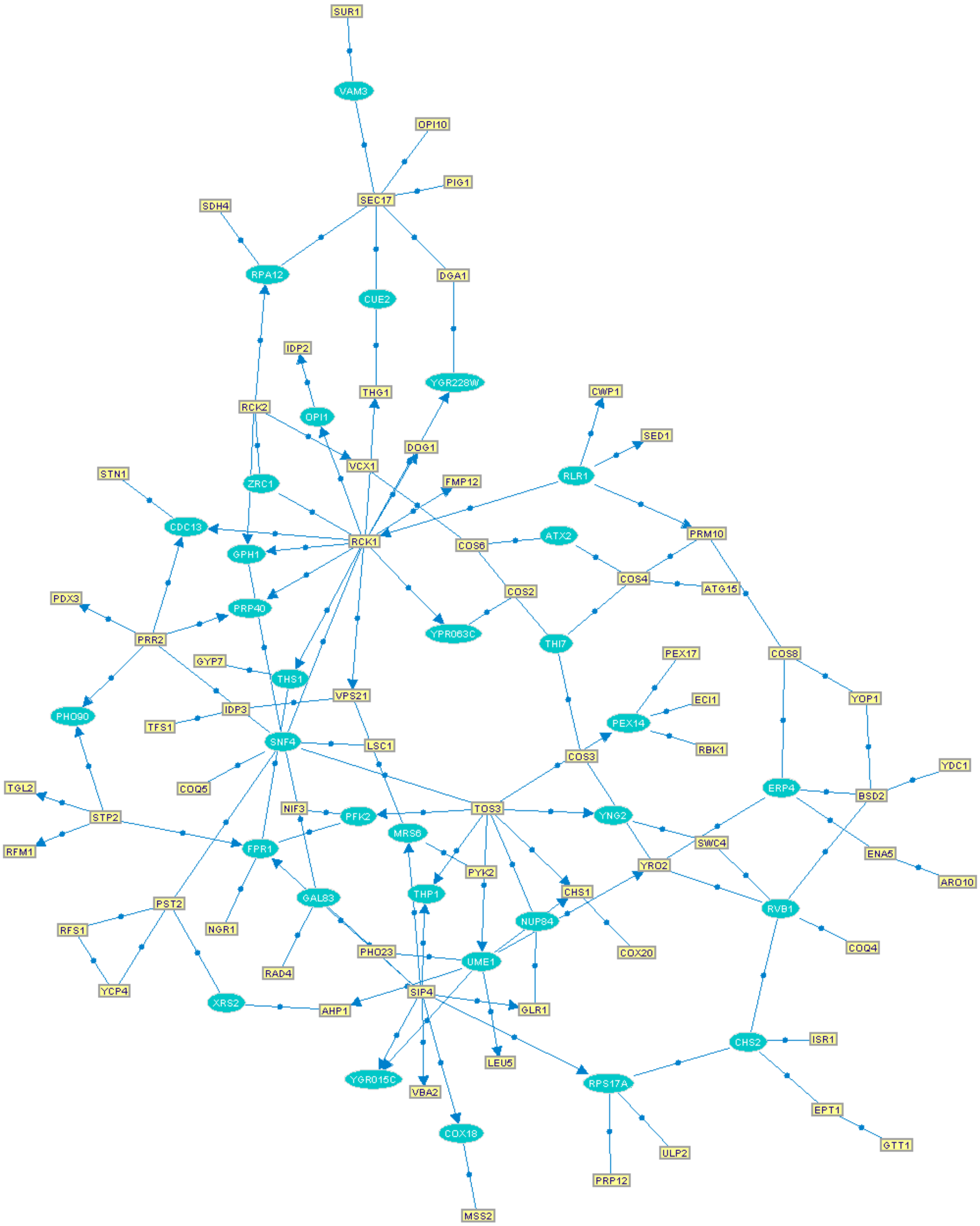

Figure 19: Osmotic shock response - JACS 19.

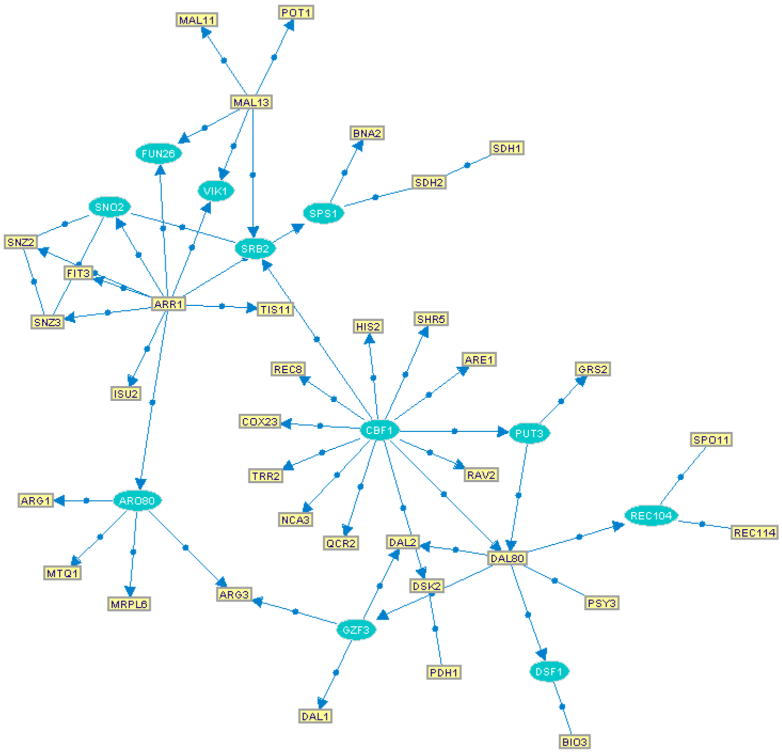

Figure 20: Osmotic shock response - JACS 20.

2 Human cell cycle

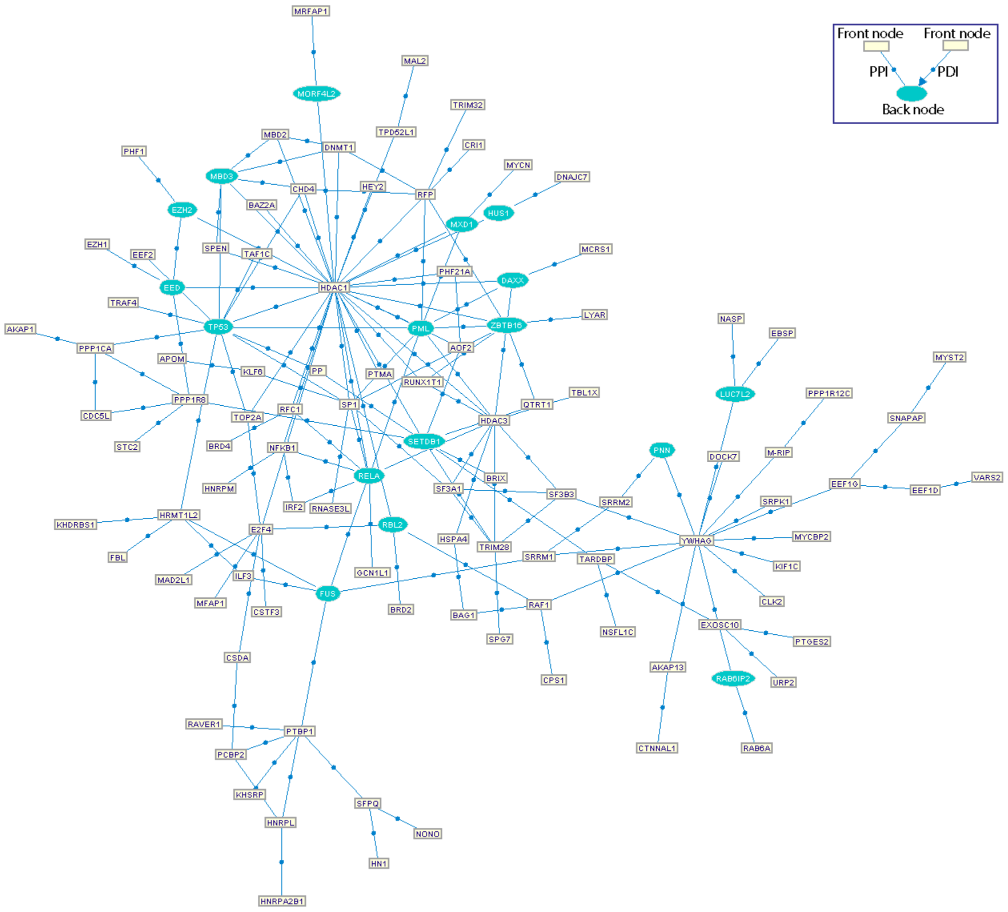

Figure 21: Human cell cycle - JACS 1.

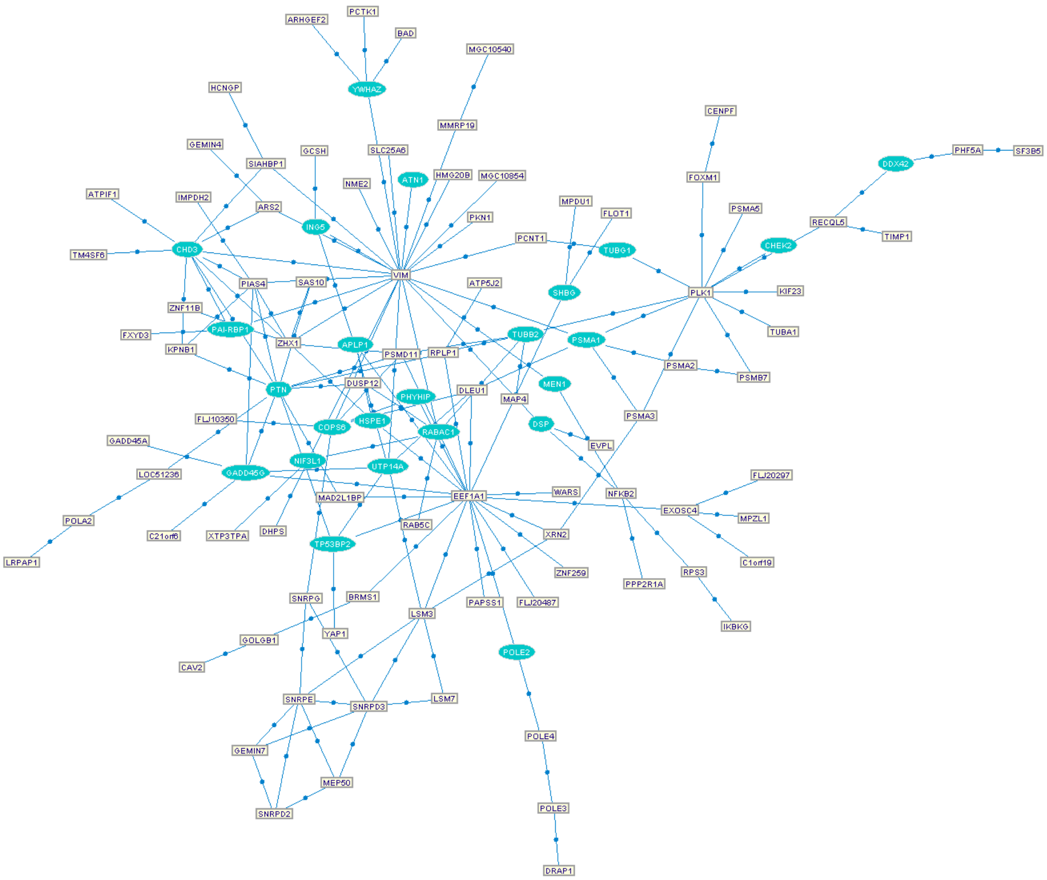

Figure 22: Human cell cycle - JACS 2.

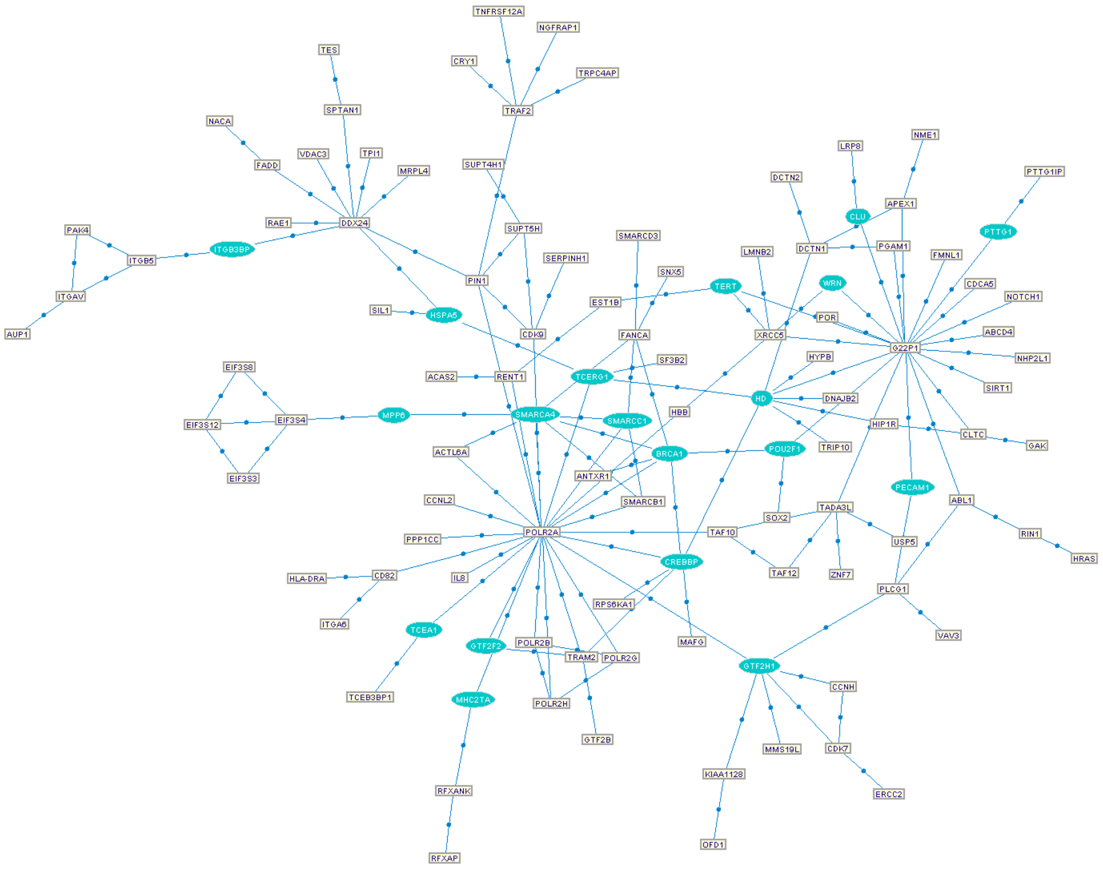

Figure 23: Human cell cycle - JACS 3.

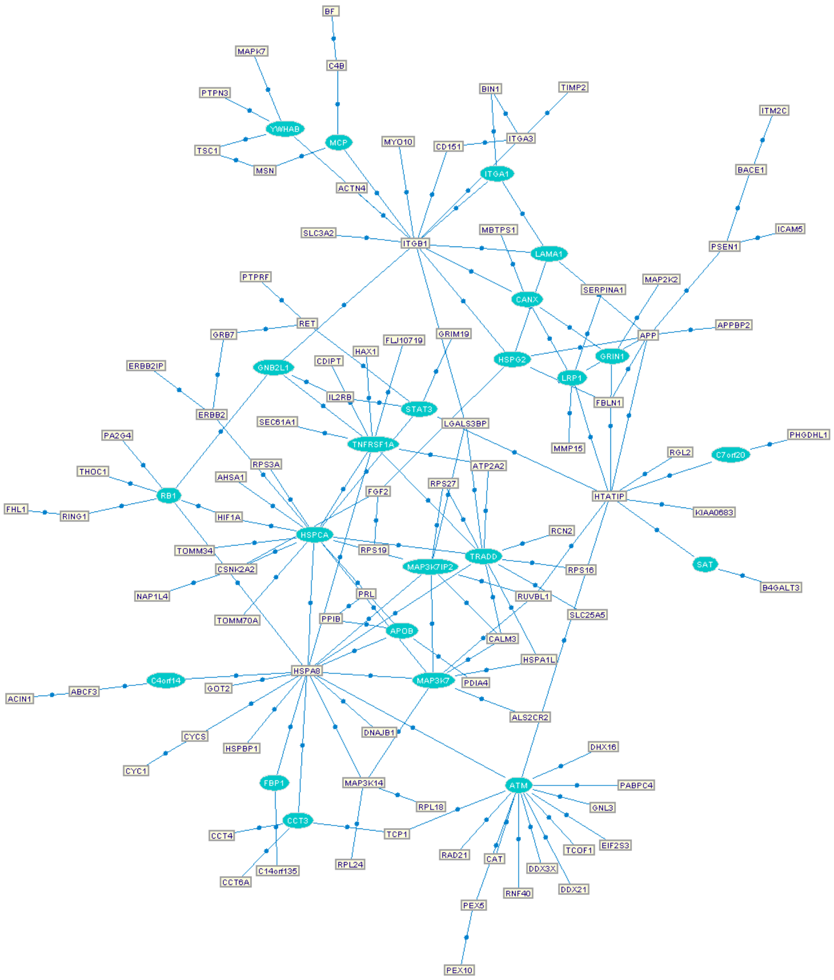

Figure 24: Human cell cycle - JACS 4.

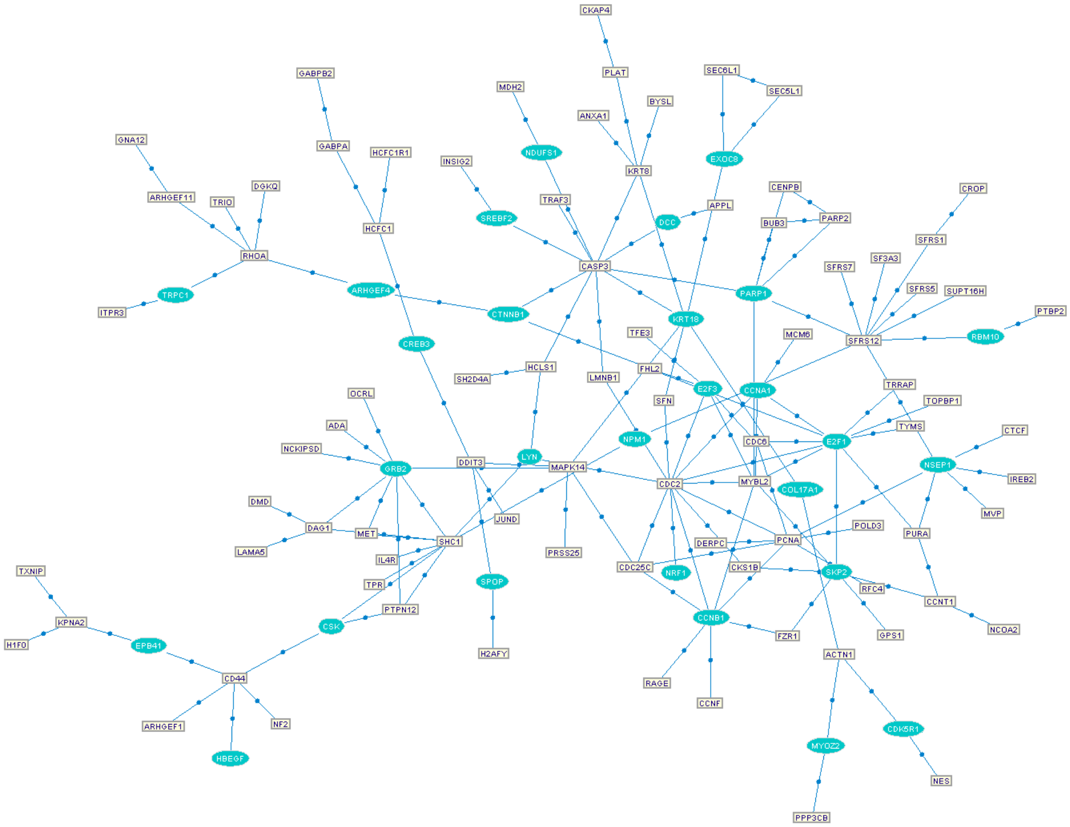

Figure 25: Human cell cycle - JACS 5.

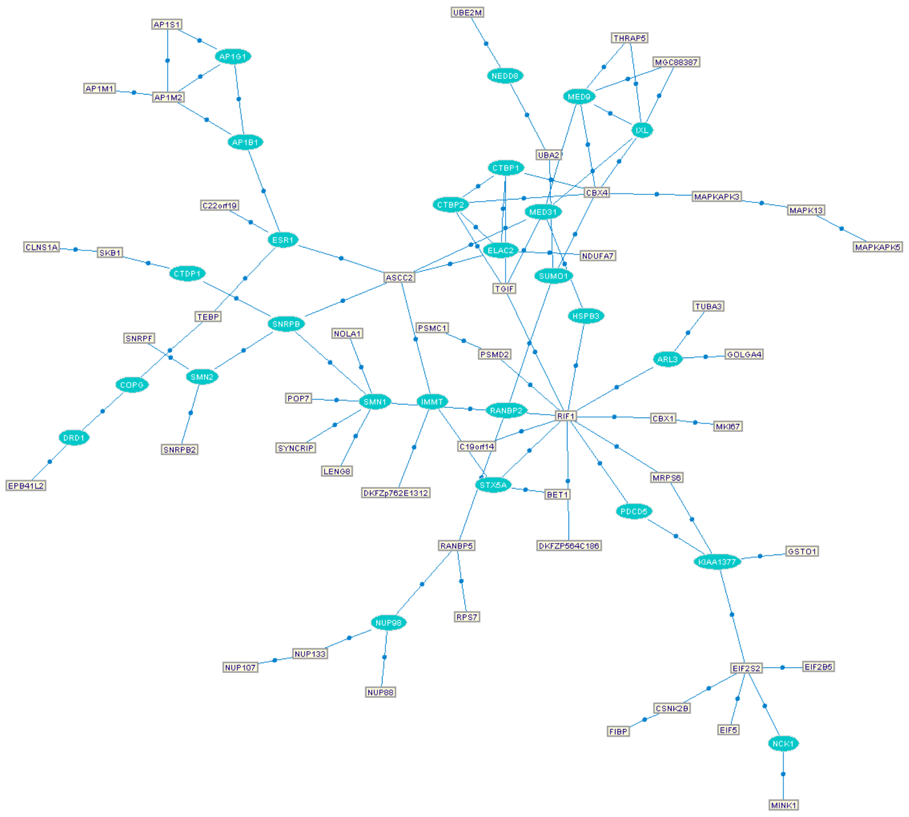

Figure 26: Human cell cycle - JACS 6.

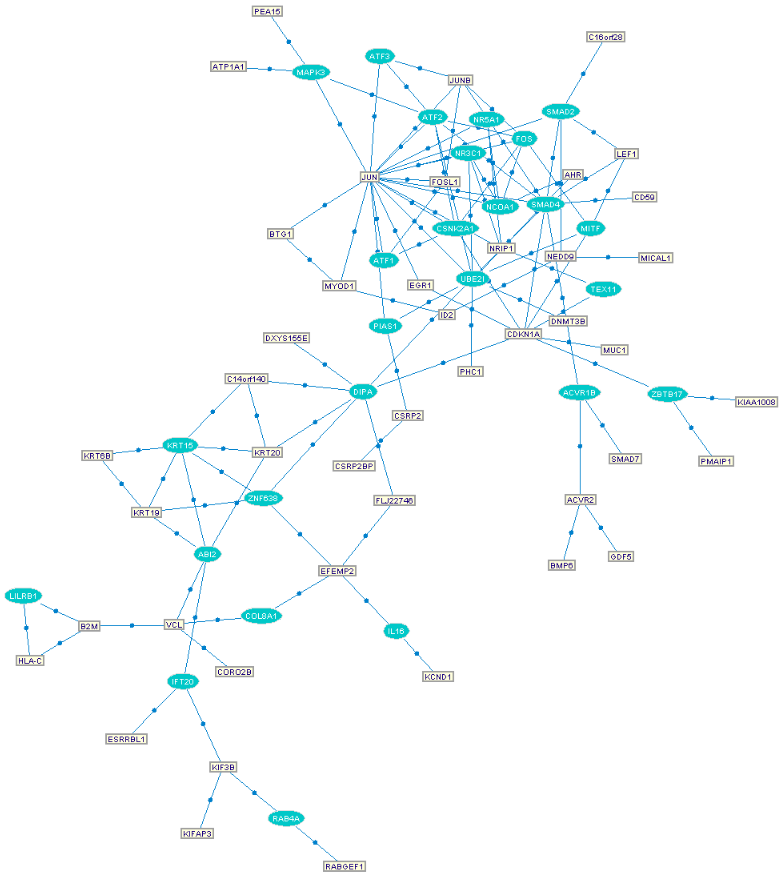

Figure 27: Human cell cycle - JACS 7.

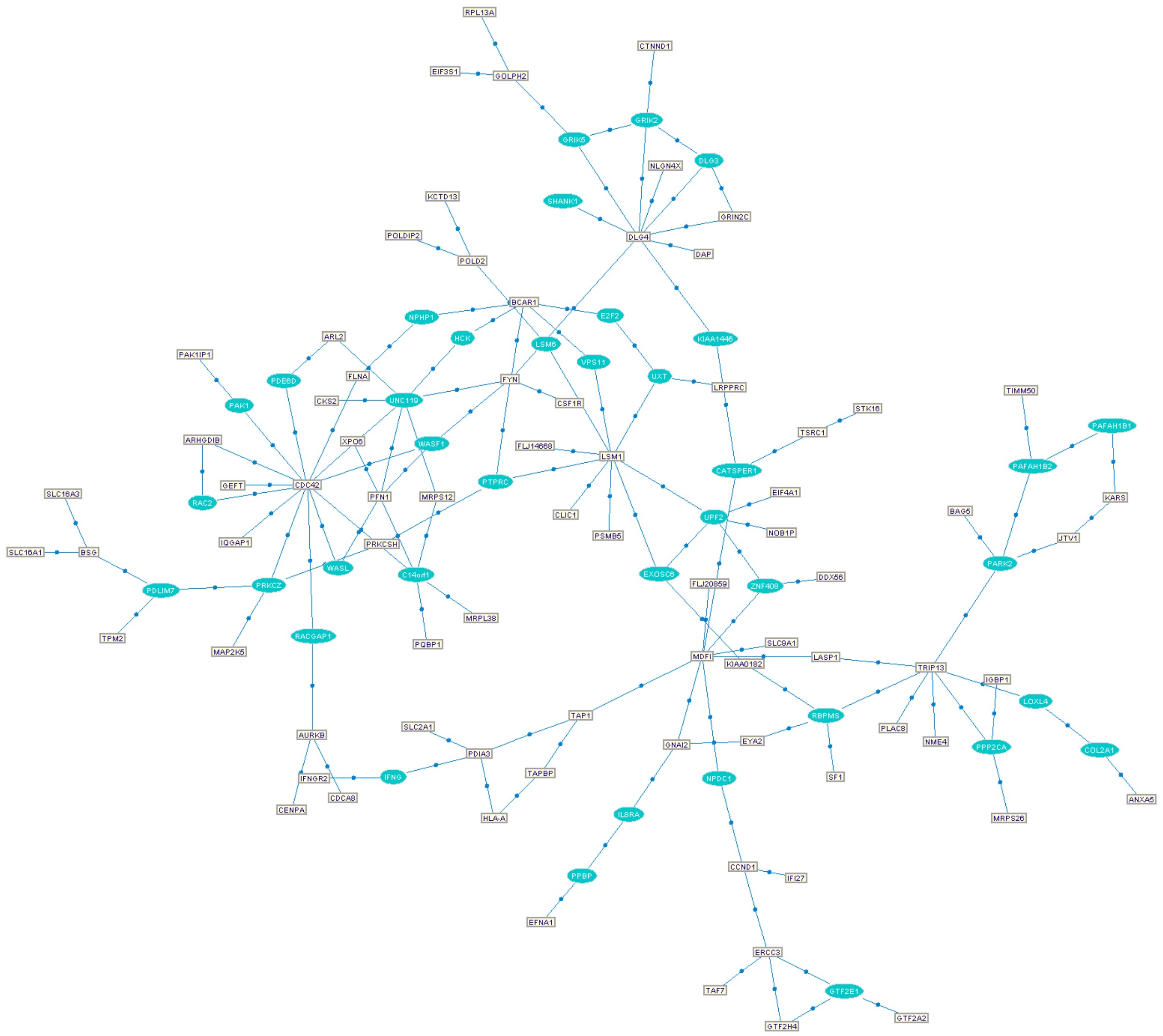

Figure 28: Human cell cycle - JACS 8.

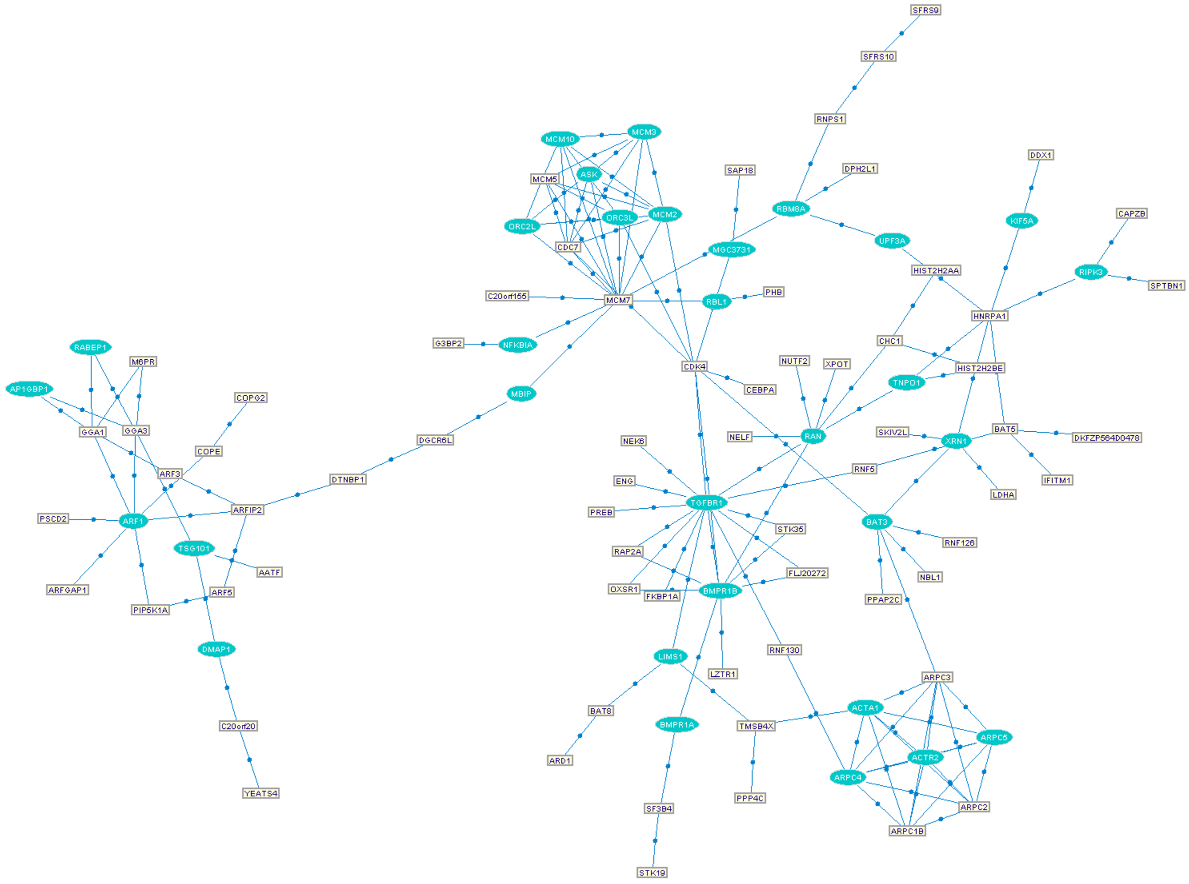

Figure 29: Human cell cycle - JACS 9.

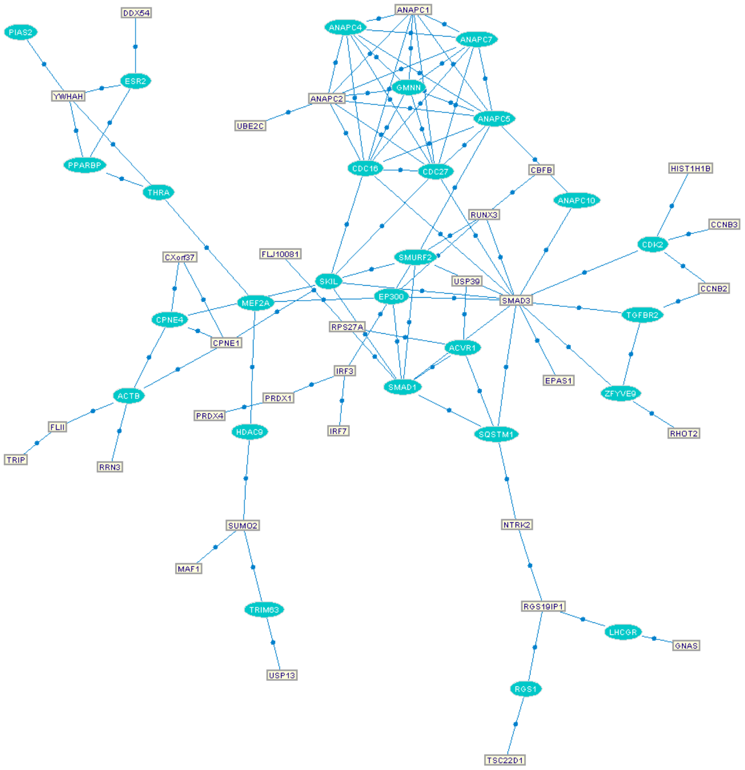

Figure 30: Human cell cycle - JACS 10.

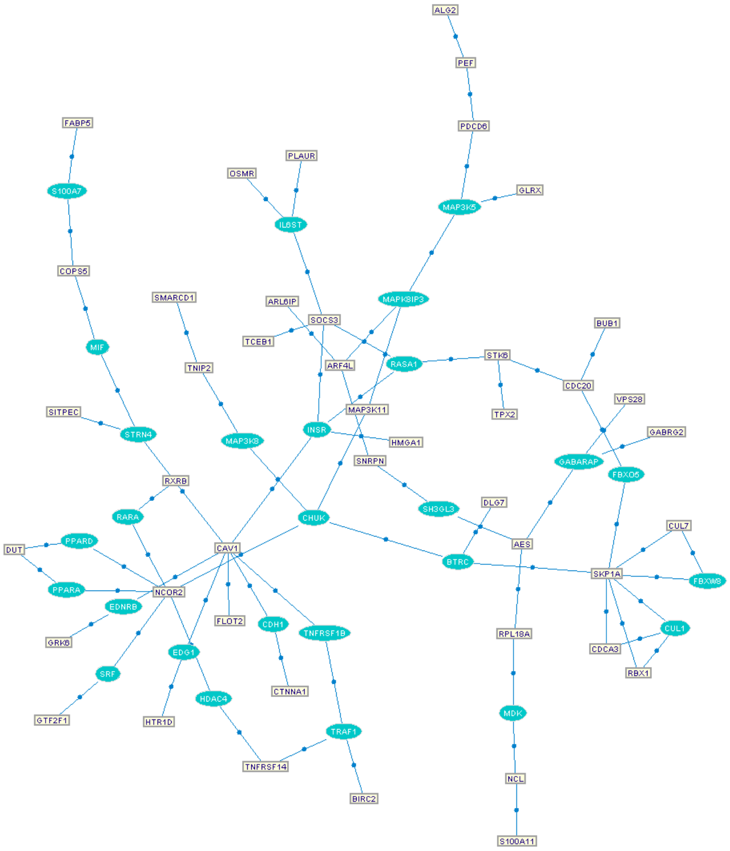

Figure 31: Human cell cycle - JACS 11.

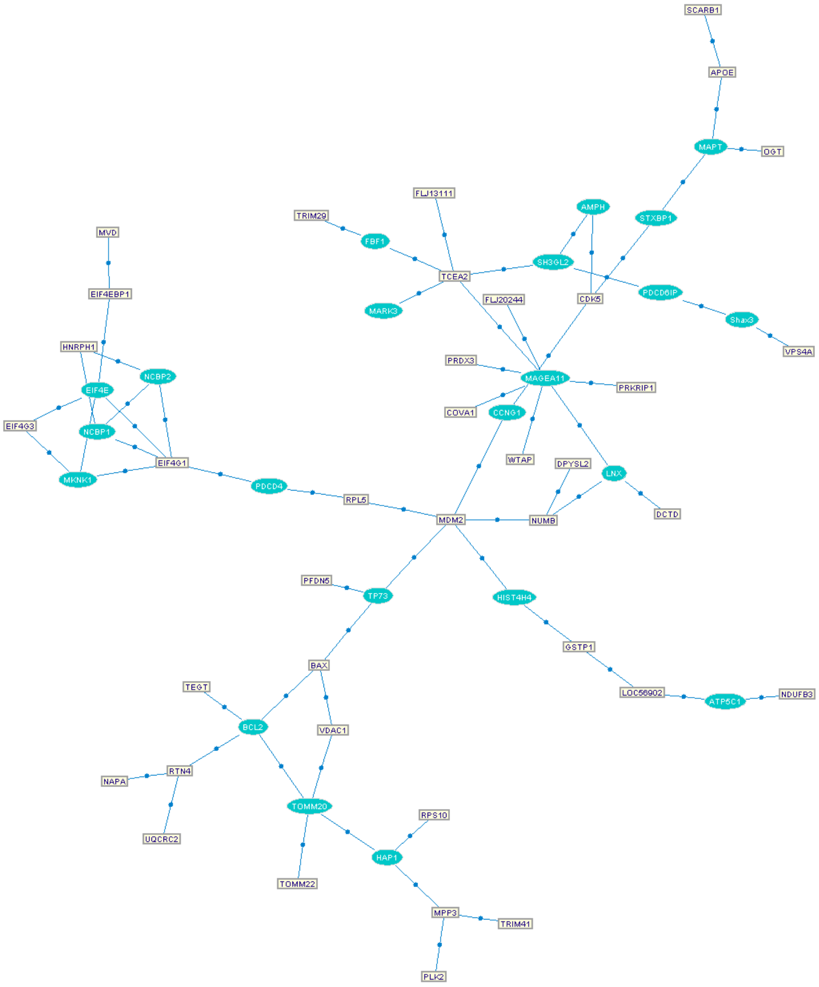

Figure 32: Human cell cycle - JACS 12.

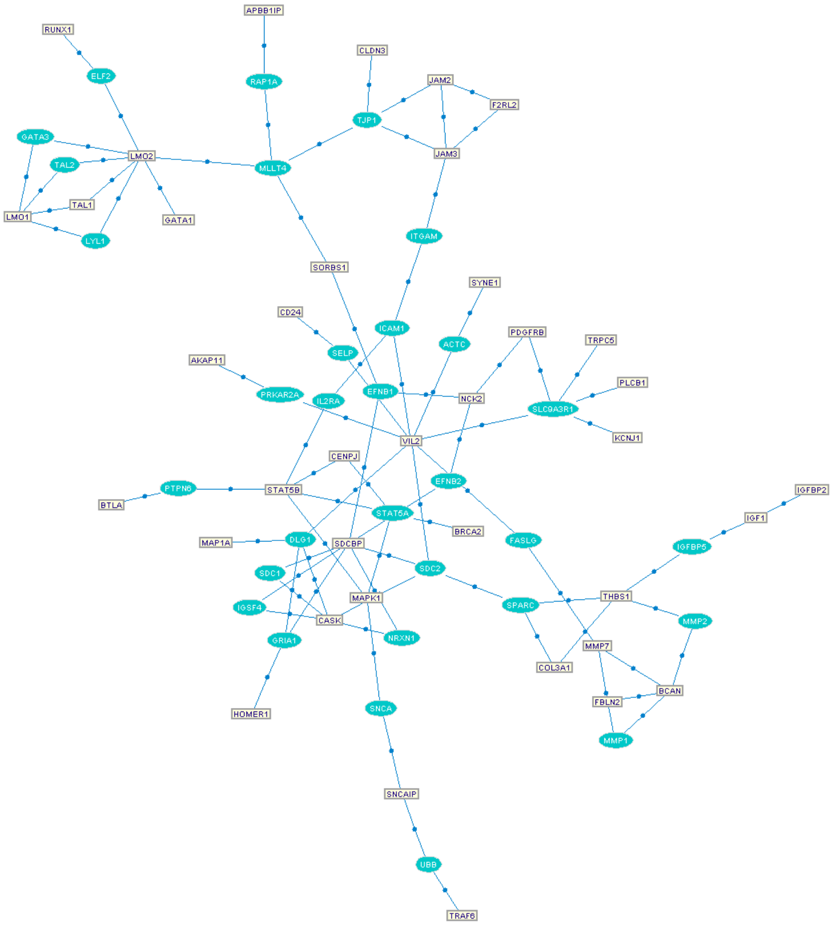

Figure 33: Human cell cycle - JACS 13.
